# Biotransformation-coupled mutasynthesis for the generation of novel pristinamycin derivatives by engineering the phenylglycine residue†

**DOI:** 10.1039/d3cb00143a

**Published:** 2023-10-14

**Authors:** Oliver Hennrich, Leoni Weinmann, Andreas Kulik, Karen Harms, Philipp Klahn, Jung-Won Youn, Frank Surup, Yvonne Mast

**Affiliations:** a Department Bioresources for Bioeconomy and Health Research, Leibniz Institute DSMZ-German Collection of Microorganisms and Cell Cultures, Inhoffenstraße 7B 38124 Braunschweig Germany yvonne.mast@dsmz.de; b Institute of Microbiology, University Stuttgart, Allmandring 31 D-70569 Stuttgart Germany; c Department Microbial Bioactive Compounds, Interfaculty Institute of Microbiology and Infection Medicine, Faculty of Science, University of Tübingen, Auf der Morgenstelle 28 D-72076 Tübingen Germany; d Microbial Drugs Department, Helmholtz-Centre for Infection Research 38124 Braunschweig Germany; e Division of Organic and Medicinal Chemistry, Department of Chemistry and Molecular Biology, University of Gothenburg, Kemigården 4 412 96 Göteborg Sweden; f Centre of Antimicrobial Resistance Research in Gothenburg (CARe) Gothenburg Sweden; g Technische Universität Braunschweig, Institut für Mikrobiologie, Rebenring 56 38106 Braunschweig Germany; h German Center for Infection Research (DZIF), Partner Site Tübingen Tübingen Germany

## Abstract

Streptogramins are the last line of defense antimicrobials with pristinamycin as a representative substance used as therapeutics against highly resistant pathogenic bacteria. However, the emergence of (multi)drug-resistant pathogens renders these valuable antibiotics useless; making it necessary to derivatize compounds for new compound characteristics, which is often difficult by chemical *de novo* synthesis due to the complex nature of the molecules. An alternative to substance derivatization is mutasynthesis. Herein, we report about a mutasynthesis approach, targeting the phenylglycine (Phg) residue for substance derivatization, a pivotal component of streptogramin antibiotics. Mutasynthesis with halogenated Phg(-like) derivatives altogether led to the production of two new derivatized natural compounds, as there are 6-chloropristinamycin I and 6-fluoropristinamycin I based on LC-MS/MS analysis. 6-Chloropristinamycin I and 6-fluoropristinamycin I were isolated by preparative HPLC, structurally confirmed using NMR spectroscopy and tested for antimicrobial bioactivity. In a whole-cell biotransformation approach using an engineered *E. coli* BL21(DE3) pET28-*hmo*/pACYC-*bcd-gdh* strain, Phg derivatives were generated fermentatively. Supplementation with the *E. coli* biotransformation fermentation broth containing 4-fluorophenylglycine to the pristinamycin mutasynthesis strain resulted in the production of 6-fluoropristinamycin I, demonstrating an advanced level of mutasynthesis.

## Abstract 2

Here, we report the development of a mutasynthesis approach for the derivatisation of pristinamycin I based on the phenylglycine residue in combination with a biotransformation process for mutasynthon provision.

## Graphical abstract

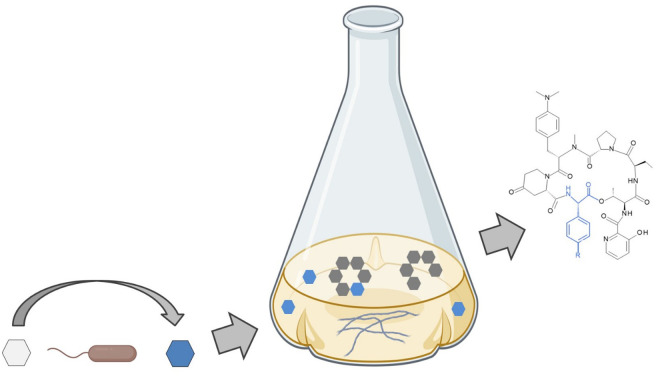

## 1. Introduction

Antibiotics are undoubtedly the most important and impactful medical advancement of all time and a cornerstone of modern medicine.^1,2^ However, the rapid emergence of (multi)drug-resistant pathogens endangers their efficacy and poses a serious threat to global human health.^3^ According to a recent estimate, more than 1.2 million people died worldwide in 2019 due to drug-resistant bacterial infections.^4^ Accompanying this development, the pipeline for new antibacterial agents was feared to run empty in recent years with only slightly encouraging rising numbers of drug candidates from 2017 onwards.^5,6^ Especially, new compound classes are rarely found. The most recent new compound classes that have been introduced to the market are cyclic lipopeptides (daptomycin by Cubist Pharmaceuticals in 2003) and oxazolidinones (linezolid by Pfizer in 2000).^7^ Thus, discovering and developing new antimicrobials and strategies to combat resistance is a key challenge in the years ahead.

The most important sources for many powerful antibiotics are actinomycetes and fungi, which are the origin of up to two-thirds of all antibiotics in clinical use today.^8^ These involve all different classes of antibiotics, such as aminoglycosides, macrolides, tetracyclines, glycopeptide antibiotics, β-lactams, rifamycins, or streptogramins.^8^ While the biosynthetic potential of actinomycetes for unknown antibiotics is still considered high,^9,10^ there are also other approaches that aim to get access to novel bioactive substances, as for instance the structural modification of already existing potent bioactive drugs.^11^ A good example of modification of a chemical scaffold is the macrolide antibiotic erythromycin, where the natural compound served as a starting point for chemical modification at the C12 position, resulting in a series of novel ketolides.^12^ All modified compounds showed high activity against Gram-positive bacteria, such as *Staphylococcus aureus*, *Streptococcus pneumoniae*, and *Streptococcus pyogenes*.^12^ The challenge in modifying such a kind of antibiotics lies in their complex chemical structures, which originate from a multistep biosynthesis. Many compounds are difficult to generate by chemical or semi-synthesis, which limits the ability to optimize compounds by chemical derivatization.^13,14^ For example, the chemical synthesis of the aglycone of the glycopeptide antibiotic vancomycin involves 19 chemical steps and is associated with several problems, notably the lack of kinetic atroposelectivity in the construction of the tricyclic scaffold, low overall yields, and the difficult synthesis of unnatural amino acids.^15,16^

**Fig. 1:**
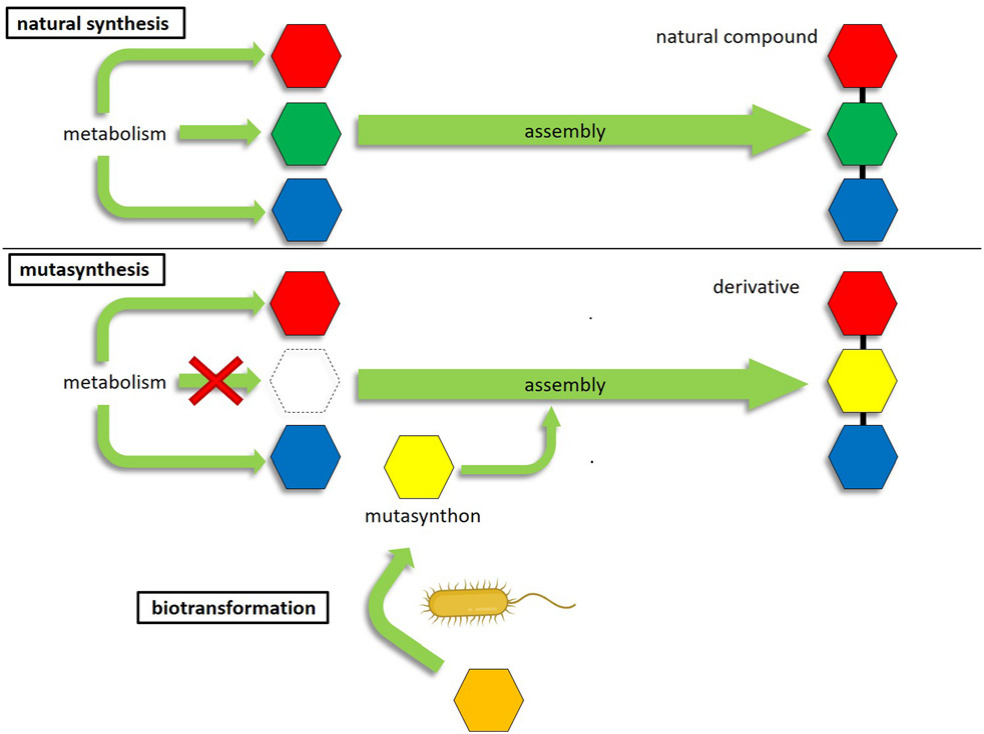
Schematic presentation of the mutasynthesis principle. Colored hexagons represent the different antibiotic building blocks, which are assembled by the biosynthetic machinery. For mutasynthesis, a block mutant is required where the synthesis of a building block is abolished. Feeding with an unnatural analog (mutasynthon (yellow hexagon)) results in the synthesis of an altered product. The provision of the mutasynthon as a result of the conversion of the precursor (orange hexagon) by the biotransformation step (bacteria symbol) is indicated below.

Genetic engineering methods are suitable alternatives for the targeted derivatization of complex natural products.^17^ These methods are based on the biotechnological manipulation of genes involved in antibiotic biosynthesis, which in bacterial antibiotic producers are usually organized in biosynthetic gene clusters (BGCs).^18^ Mutasynthesis is a method of metabolic engineering that combines aspects of chemical synthesis with genetic engineering techniques (= mutational biosynthesis). The principle of mutasynthesis involves the genetic disruption of one or more gene(s) responsible for the biosynthesis of a key precursor, with subsequent supply of synthetic analogues of the missing precursor, designated as mutasynthons. The incorporation of a mutasynthon into the biosynthetic assembly line of the secondary metabolite then leads to the formation of novel compound derivatives (Fig. 1).^19^ In this way, mutasynthesis combines genetic manipulation with the principle of precursor-directed biosynthesis^20^ to exploit substrate promiscuity in the biosynthetic assembly lines of natural product producers.^13^ This can be used to modify complex natural products that are inaccessible or difficult to access by means of chemical synthesis.^13,14,21^ The advantage of mutasynthesis over precursor-directed biosynthesis is that by using a precursor block mutant, there is no competition between the natural precursor and the unnatural mutasynthon building block, resulting in the biosynthesis of the desired mutasynthesis product only. A prerequisite for this powerful technology is the knowledge of the biosynthetic pathway, especially that the respective precursor genes are known, the genetic tractability of the antibiotic producer strain, as well as that the mutasynthon is readily taken up by the cell and accepted by the biosynthetic machinery.^19,22–24^ Mutasynthesis has been successfully applied for the derivatization of various compound classes, such as polyketides,^25–27^ nonribosomal peptides^28^ glycopeptide antibiotics,^14^ aminoglycosides,^29^ alkaloids,^30^ aminocoumarins,^31^ or lincomycins.^32^ For example, regarding drug optimization, mutasynthesis has successfully been applied to generate derivatives of lincomycin, which showed increased activities against low-level lincosamide resistant staphylococci compared to the original compound.^32^ Another example includes a series of novel physostigmine alkaloids generated by mutasynthesis, where one of the modified compounds exhibited an improved selectivity and toxicity profile.^30^

**Fig. 2:**
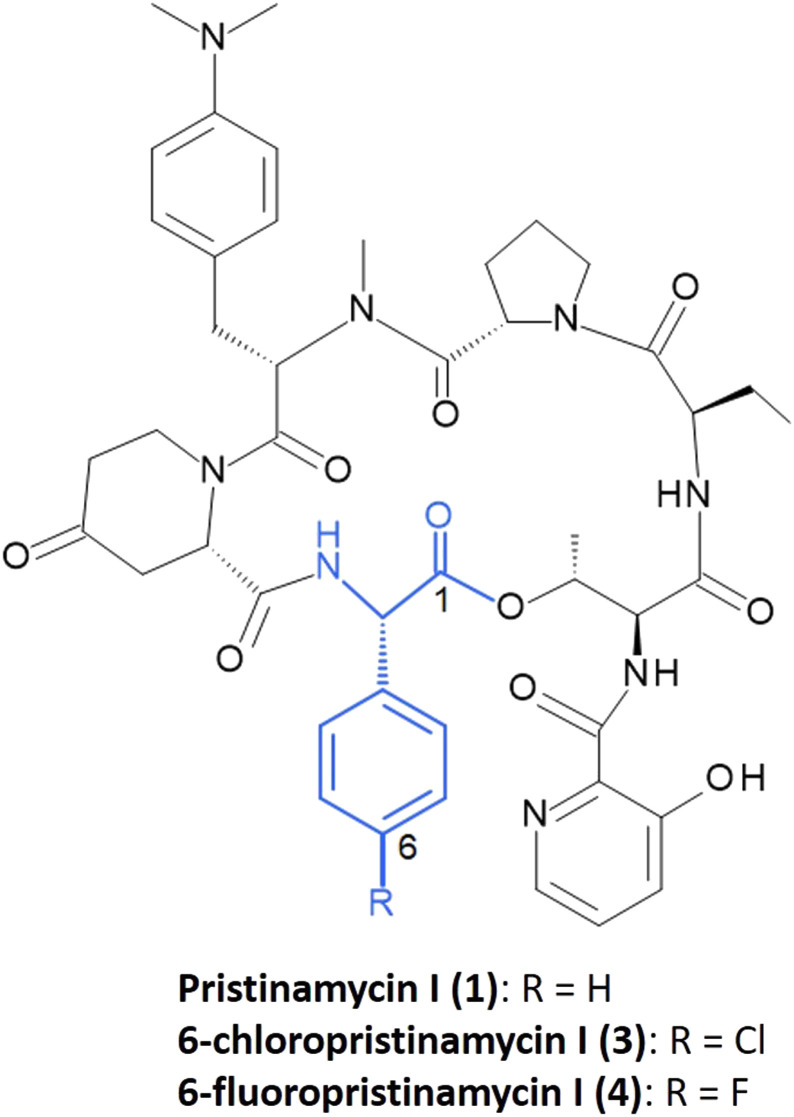
Chemical structure of 1, 3, and 4 with Phg residue indicated in blue. Numbering indicates the order of incorporation into the peptide backbone.

**Fig. 3:**
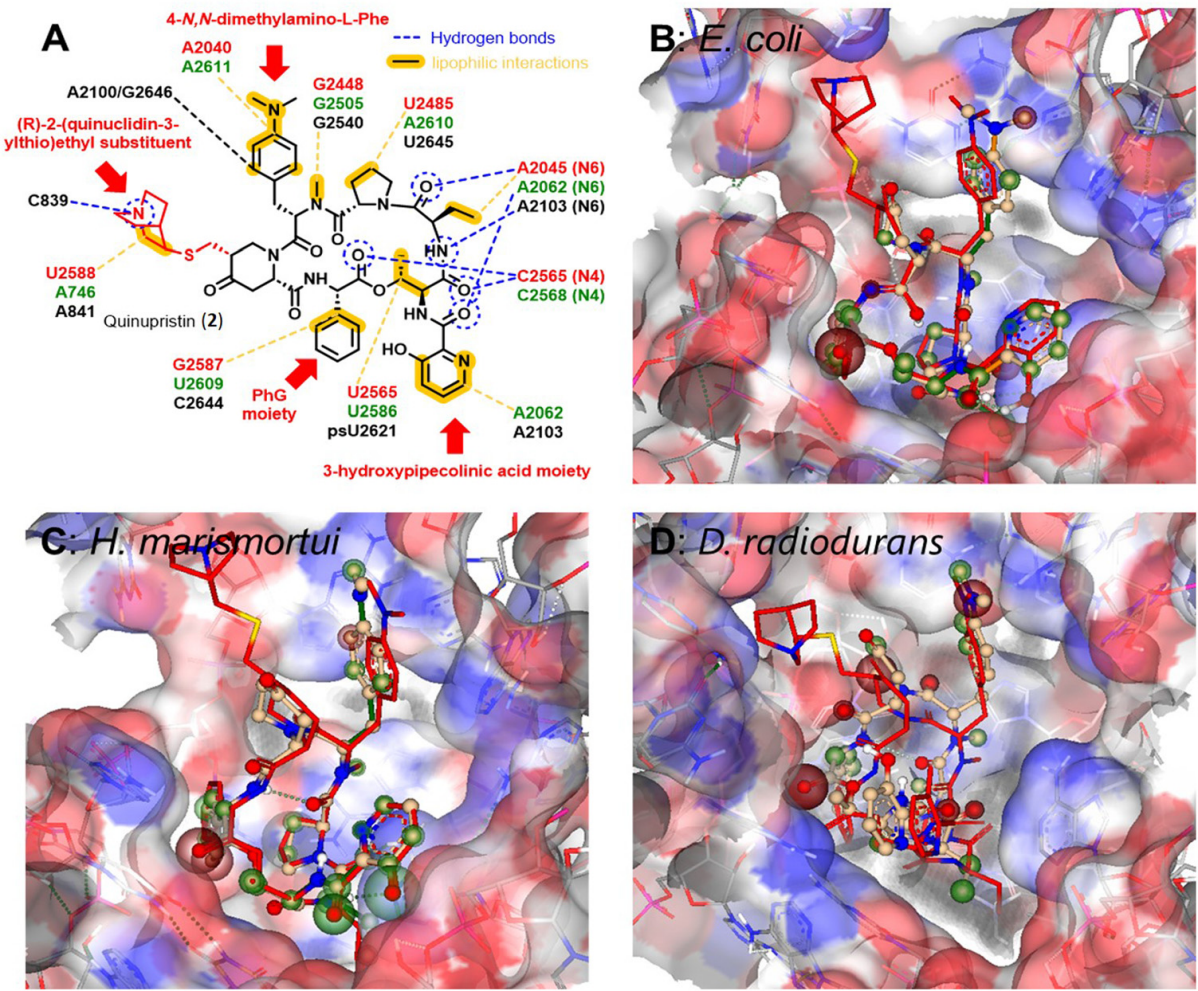
(A) Structure of quinupristin (2) and illustration of the network of hydrogen bonds and lipophilic interactions with bacterial 23S rRNA bases in the 50S subunit of the bacterial ribosomes (green: *E. coli*, PBD: 4U26,^44^ red: *Deinococcus radiodurans*, PBD: 1SM1,^35^ Black: *Haloarcura marismortui*, PBD: 1YJW.^34^ Illustration of docked pristinamycin (1) into the binding pocket at the 23S rRNA of *E. coli* (B), *H. marismortui* (C) and *D. radiodurans* (D) in comparison to quinupristin (2, depicted in red) prepared with SeeSAR software version 13.0.1 (https://www.biosolveit.de/SeeSAR). Colored spheres around atoms indicate positive (green) or negative (red) contributions to the overall binding affinity based on ligand–target interactions as well as desolvatization energies.

Streptogramin antibiotics are a substance class that with pristinamycins (Synercid®, Pyostacin®) have found their way into clinical applications. Pristinamycins are used as an antibiotic of last resort to treat multidrug-resistant bacterial infections.^33^ These antibiotics show good activities against numerous Gram-positive pathogens, including methicillin- and vancomycin-resistant *Staphylococcus aureus* (MRSA, VRSA) and vancomycin-resistant *Enterococcus faecium* strains (VREF). Despite the longstanding use of pristinamycin for more than 50 years, especially in French-speaking countries, the frequency of resistance remained low but has increased in recent years,^33^ which makes it necessary to optimize the substance in terms of broad-spectrum activity and resistance breaking characteristics, which can be achieved by derivatization of the compound by mutasynthesis. However, so far mutasynthesis has been prevented by the lack of information about precursor biosynthesis and its encoding genes. In particular, the genes coding for the biosynthesis of the non-proteinogenic amino acid l-phenyglycine (l-Phg), which is a constituent of the peptide antibiotic pristinamycin I (1), were not known until recently.

l-Phg is essential for the bioactivity of streptogramin antibiotics, such as pristinamycin I (1) or its semi-synthetic derivative Synercid®.^34,35^ Pristinamycin is a natural compound mixture produced by *Streptomyces pristinaespiralis* and consists of a mixture of two types of chemically unrelated substances – the macrocyclic polyketide/nonribosomal peptide hybrid compound pristinamycin II (= streptogramin group A (S_A_)) and the cyclic hexadepsipeptide pristinamycin I (1) (= streptogramin group B (S_B_)), the latter containing Phg as a structural component (Fig. 2).^36^ Pristinamycin I and II are co-produced by *S. pristinaespiralis* in a synergistically active ratio of 30 : 70.^36^ Both compounds inhibit the bacterial protein biosynthesis by binding to the 23S rRNA of the 50S subunit of bacterial ribosomes, whereby pristinamycin II prevents the binding of the aminoacyl-tRNA, while pristinamycin I (1) causes the dissociation of the peptidyl-tRNA from the ribosome.^35^ Each component alone exhibits moderate bacteriostatic activity, whereas in combination they act synergistically, resulting in a potent bactericidal activity.^35^ The synergistic mixture is used for clinical application, *e.g.* as Synercid®, which is a combination of semi-synthetic derivatives of pristinamycin I (quinupristin (2), Fig. 3) and pristinamycin II (dalfopristin).^35^ Resistances against streptogramin antibiotics include, *e.g.* the active efflux of the antibiotics or the enzymatic inactivation of either of the two components.^36^ The most prevalent resistance mechanism against S_B_ antibiotics is the *N*^6^ (di)methylation of a specific adenine nucleotide residue (A2058, *Escherichia coli* numbering) of the ribosomal 23S rRNA.^37,38^ This modification is mediated by Erm methyltransferases, encoded by plasmid-borne *erm* genes, which leads to a drastic reduction of affinity for members of the macrolide-lincosamide-streptogramin B (MLS_B_) group of antibiotics.^39^ In *S. pristinaespiralis*, the S_B_ antibiotic 1 is synthesized by the nonribosomal peptide synthetases (NRPSs) SnbA, SnbC, and SnbDE, whereby the latter one incorporates l-Phg as the final amino acid into the growing pristinamycin I peptide chain.^36,40^l-Phg biosynthesis occurs through a series of enzymatic reactions, catalyzed by the enzymes phenylpyruvate dehydrogenase (PglB/PglC), Phg dehydrogenase (PglA), thioesterase (PglD), and aminotransferase (PglE), which convert phenylpyruvate to l-Phg.^18,41,42^ The genetic information for Phg biosynthesis is encoded on the *S. pristinaespiralis pglA-E* operon.^41^ So far, this is the first reported Phg biosynthetic pathway and has been identified in the frame of characterizing the pristinamycin biosynthetic gene cluster.^40,41,43^ Crystallographic analysis of a ribosome-bound streptogramin antibiotic revealed the formation of a hydrogen bond between the l-Phg carbonyl oxygen with nucleotide C2586 of the 23S rRNA (*E. coli* numbering) as part of the interactions involved in the binding of 1 to the ribosome.^35^ Additionally, the aromatic ring of Phg is involved in establishing hydrophobic interactions with the ribosome^34^ hinting at the functional importance of the amino acid for bioactivity.

The pristinamycin biosynthetic route is well-described and the producer strain *S. pristinaespiralis* is genetically tractable,^40^ making it strain a suitable chassis strain for a streptogramin mutasynthesis approach. In the current study, we report on a mutasynthesis approach for the derivatization of pristinamycin I (1) by targeting the Phg-like residue for modification to generate novel antibiotic derivatives.

## 2. Results and discussion

### 2.1 Docking of pristinamycin into the ribosomal binding pocket reveals options for pristinamycin I derivatization

In order to estimate the potential for compound derivatisation, computational docking studies were carried out with known pristinamycin compounds and bacterial ribosomes as drug targets. Quinupristin (2), another clinically applied pristinamycin I derivative bearing an additional (*R*)-2-(quinuclidin-3-ylthio)ethyl substituent at the 4-oxo-l-pipecolic acid moiety (Fig. 3A), has been co-crystallized with the ribosome of different species including *E. coli* and the two extremophiles *Deinococcus radiodurans* and *Haloarcura marismortui*.^34,35,44^ The structure analysis of these co-crystals reveals that quinupristin (2) binds the 23S rRNA of the bacterial large ribosomal subunit 50S at the entrance of the ribosomal tunnel through a network of hydrophobic interactions predominantly mediated and hydrogen bonds between the N6 nitrogen of the A2062 nucleotide (*E. coli nomenclature in green*) and the N4 nitrogen of the C2565 nucleotide (*E. coli nomenclature in green*) as outlined in Fig. 3A^34,35,44^ with no direct contact to the ribosome protein backbone. Pristinamycin I (1) is believed to bind in a similar manner to the same binding pocket at 23S rRNA of the bacterial ribosomal 50S subunit. However, as no co-crystal structure of 1 with the bacterial ribosome is available, we have performed a virtual docking of pristinamycin I (1) into the binding pocket of quinupristin (2) at the ribosome based on the three available co-crystal structure with the ribosome of *E. coli* (PDB: 4U26), *D. radiodurans* (PDB: 1MS1), and *H. marismortui* (PBD: 1YJW)^34,35,44^ using the software SeeSAR (SeeSAR version 13.0.1; BioSolveIT GmbH, Sankt Augustin, Germany, 2023, www.biosolveit.de/SeeSAR). The general overlap of pristinamycin (1) with quinupristin (2) in cases *E. coli* (Fig. 3B, see PDB file 3B_*E. coli* in the ESI†) and *H. marismortui* (Fig. 3C, see PDB file 3C_*H. marismorturi* in the ESI†) is very high, with only very little deviations especially in the position of the 4-*N*,*N*-dimethylamino-l-Phe side chain and the 3-hydroxypipecolinic acid moiety. In the case of *D. radiodurans* (Fig. 3D, see PDB file 3D_*D. radiodurans* in ESI†) already 2 is bound in a slightly different orientation compared to *E. coli* and *H. marismortui*, in particular, the 3-hydroxypipecolinic acid moiety is oriented to the opposite direction. The docked structure of 1 is then shifted by approximately 2.1 Å into the binding pocket of the 23S rRNA compared to 2. Presumably, this is a consequence of lacking the (*R*)-2-(quinuclidin-3-ylthio)ethyl substituent of 2 allowing 1 to enter deeper into the pocket. The Phg moiety of 1 points in all cases towards the inside of the 23S rRNA binding pocket. However, unoccupied space between the 23S rRNA nucleotides and the Phg moiety in all docking approaches encouraged us to virtually explore further possible Phg derivatives, which could be accessed through mutasynthesis *via* an *in silico* screening of 4-, 3- and 2-substituted Phg derivatives based on the docking of 1 into the binding pocket of *E. coli*. In general, smaller substituents in the 3- and 4-position seemed to be tolerated for binding to the target. Substitution in the 2-position of the Phg moiety mostly led to derivatives presumably not binding to the target structure anymore.

### 2.2 Construction of a biosynthetic mutant of *S. pristinaespiralis* for mutasynthesis

**Fig. 4:**
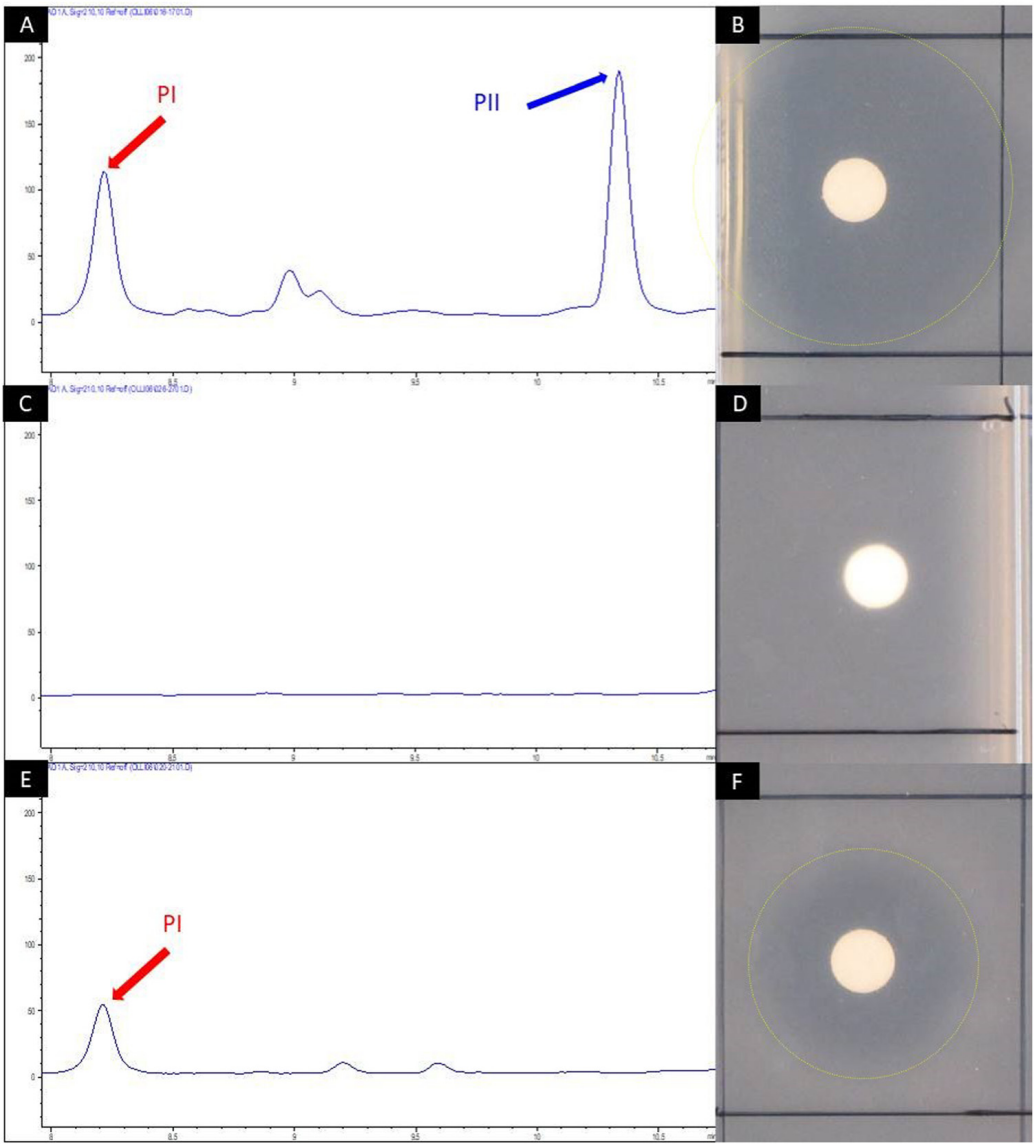
HPLC chromatogram of crude extract samples from *S. pristinaespiralis* wild-type (A), *S. pristinaespiralis ΔpglAΔsnaE1* (C) and *ΔpglAΔsnaE1* supplemented with 100 μM l-Phg (E). The chromatogram shows peaks measured at 210 nm. Pristinamycin I (PI) and II (PII)-specific peaks were detected at retention times of 8.2 and 10.35 min and are indicated by red and blue arrows, respectively. Disc diffusion assays with the respective extract samples against *B. subtilis* (B), (D) and (F). Inhibition zones are marked with yellow circles.

To allow for the mutasynthesis of Phg-containing pristinamycin I derivatives, an appropriate biosynthetic mutant deficient in Phg precursor supply had to be generated. In *S. pristinaespiralis*, *pglA* is the first gene of the l-Phg biosynthesis encoding operon *pglA-E* and has been shown to be essential for pristinamycin I production.^41^ Inactivation of *pglA* in *S. pristinaespiralis* (M*pglA*) resulted in a loss of pristinamycin I production, whereas feeding *MpglA* with l-Phg restored pristinamycin I production, thus functionally complementing the *pglA* inactivation.^41^ The production of the S_A_ antibiotic pristinamycin II is not affected in the *MpglA* mutant.^41^ To obtain an *S. pristinaespiralis* mutasynthesis strain that produces pristinamycin I derivatives exclusively, we aimed to abolish the biosynthesis of the pristinamycin II compound as well. This should allow for PI derivative production in an antibiotic-free background so that the mutasynthesis samples can be directly tested for bioactivity. For this purpose, the gene *snaE1*, encoding a hybrid PKS/NRPS enzyme essential for pristinamycin II biosynthesis^45^ was inactivated by insertion of a thiostrepton resistance cassette (*thio*^*R*^) using the mutational plasmid pK18-*snaE1tsr*, which resulted in the double mutant *S. pristinaespiralis ΔpglAΔsnaE1* (Table S1, ESI†). The *ΔpglAΔsnaE1* mutant and the *S. pristinaespiralis* wild-type strain (control) were cultivated in pristinamycin production medium HT7T, respectively. After 72 h of cultivation, the culture extracts were analyzed for pristinamycin production by HPLC. The HPLC spectrum of the *S. pristinaespiralis* wild-type (WT) extract showed specific peaks at retention times (RT) 8.2 min and 10.35 min (Fig. 4A) with characteristic pristinamycin I and II UV-Vis spectra, respectively (data not shown). In addition, the WT extracts exhibited bioactivity against *B. subtilis* (Fig. 4B). In contrast, such a HPLC peak pattern was not detected for the *S. pristinaespiralis ΔpglAΔsnaE1* extract samples (Fig. 4C) and extracts did not exert antibiotic activity against *B. subtilis* (Fig. 4D). These results confirmed that the *S. pristinaespiralis ΔpglAΔsnaE1* mutant was no longer capable of synthesizing pristinamycin I nor pristinamycin II. Extract samples obtained from *S. pristinaespiralis ΔpglAΔsnaE1* cultures, supplemented with l-Phg revealed a single peak at RT 8.2 min in HPLC analysis (Fig. 4E) and exerted moderate bioactivity against *B. subtilis* (Fig. 4F), which showed that pristinamycin I production was restored exclusively. These results proved the functional correctness of the *S. pristinaespiralis ΔpglAΔsnaE1* mutant and enabled its use as a mutasynthesis strain for Phg-directed derivatisation of pristinamycin I.

### 2.3 Generation of novel pristinamycin I derivatives by mutasynthesis

To conduct the mutasynthesis of pristinamycin I, a series of commercially available Phg derivatives was used as mutasynthons for feeding experiments with *S. pristinaespiralis ΔpglAΔsnaE1* (Table 1). These included a variety of halogenated and non-halogenated Phgs substituted at different positions of the aromatic residue, as well as enantiomers. The mutasynthons were added to the growing cultures of *S. pristinaespiralis ΔpglAΔsnaE1*, and ethyl acetate crude extract from *S. pristinaespiralis* cultures was analyzed for the production of pristinamycin I derivatives by HPLC and HPLC/MS. For *S. pristinaespiralis ΔpglAΔsnaE1*, feeding with six mutasynthons, namely d-Phg, 2,5-dihydro-d-Phg, 4-fluoro-l-Phg, 2-fluoro-dl-Phg, 4-chloro-dl-Phg, and hydroxy-dl-Phg led to the production of the respective pristinamycin I derivatives, which were identified by HPLC-MS analysis based on their expected calculated masses (Table 1). Thereof, extract samples of *S. pristinaespiralis ΔpglAΔsnaE1* cultures supplemented with d-Phg, 4-fluoro-l-Phg, and 4-chloro-dl-Phg showed activity in bioassays against the test organism *B. subtilis* (Table 1). Because of the comparatively stable and good antibiotic production behavior of the *ΔpglAΔsnaE1* mutant when supplemented with 4-fluoro-l-Phg, and 4-chloro-dl-Phg, further efforts were focused on the isolation of the two halogenated pristinamycin I derivatives, which were referred to as 6-fluoropristinamycin I (3) and 6-chloropristinamycin I (4), respectively. Notably, feeding of d-Phg also led to the production of a pristinamycin I derivative as identified by HPLC-MS analysis, although the enantiomeric l-Phg is the natural precursor of pristinamycin I biosynthesis. Here, it remains unclear whether the adenylation domain of SnbE is able to accept d-Phg as a substrate or whether d-Phg was converted to l-Phg prior to incorporation. Since Phgs were solubilized by using sodium hydroxide prior to addition to the culture, chemical racemisation to d/l-Phg mixtures is quite possible. Though, polarimetric analysis of l- and d-Phg under alkaline conditions did not indicate racemisation (data not shown), which however does not exclude racemization in the organism. In a previous study, it has been described that the thioesterase domain of SnbDE can only accept l-Phg but not d-Phg,^46^ which makes incorporation of d-Phg in PI unlikely. Natural pristinamycin I (produced from *S. pristinaespiralis ΔpglAΔsnaE1* supplemented with l-Phg) was used as a control and was referred to as C-PI. Production of 3 and 4 (for structure see Fig. 2) was confirmed using HPLC-MS/MS analysis by comparing the fragmentation pattern of 1 with that of the respective halogenated derivatives (Fig. S1, ESI†). Three prominent, reoccurring mass signals (*m*/*z* = 578.3, 663.3, and 839.4) were detected and assigned to fractions of the parental ion pristinamycin I (Fig. S1a, ESI†). These fragments, which were determined to include the l-Phg residue in pristinamycin I, were shifted in masses corresponding to the addition of the respective halogen atom introduced (+34 for the chlorine main isotope, +18 for fluorine; shift is the mass of the atom −1 to account for the mass of the replaced hydrogen (Fig. S1b and c, respectively, ESI†)), suggesting the incorporation of the respective halogenated mutasynthons at the expected position in pristinamycin I. In order to produce sufficient amounts of substance for the structural elucidation of 3 and 4 by NMR analysis, mutasynthesis was carried out on a large scale. In total, 12 l and 6 l cultures were extracted for 3 and 4 isolation, respectively. HPLC fractions were analyzed by HRESIMS analysis to confirm the presence of 3 and 4 (Fig. S2 and S3, ESI†), respectively, whereby HRESIMS data of 1 served as a reference (Fig. S4, ESI†). HPLC fractions containing 0.6 mg of 3 and 1.54 mg of 4, respectively, were subjected to NMR analysis (NMR data for 1, 3, and 4 are available in Table 2 and in ESI,† Fig. S5–S21). As expected, the NMR data of 3 were highly similar to those of 1 (Table 2). However, the presence of two broad doublets (*δ*_H_ 7.24 and 7.37) in the ^1^H NMR spectrum as part of the characteristic AA′BB′ system indicated the incorporation of the 4-chloro-Phg unit. In the ^13^C NMR spectrum of 4, the splitting of the signals C-4/C-8, C-5/C-7 and C-6 with coupling constants *J*_C,F_ = 8.6 Hz, 21.5 Hz and 245 Hz, respectively, indicated the coupling to the fluorine atom. Furthermore, the single peak at *δ*_F_ = −113.4 ppm gave direct proof of fluorine incorporation (Table 2 and Fig. S22, ESI†). The production rates of the PI derivatives were difficult to determine due to the inconsistent production behavior of the *ΔpglAΔsnaE1* mutant. PI derivative concentrations were only measured for the pooled samples of C-PI, 3, and 4, which were produced in amounts of 1.52 μg mL^−1^, 1.55 μg mL^−1^, and 1.51 μg mL^−1^, respectively. Thus, the data indicate an incorporation of the unnatural derivatives at almost the same efficiency as the natural l-Phg precursor. Furthermore, the production of C-PI reached roughly half the amount produced by the wild-type strain (Fig. 4), which yielded an average of about 5 μg mL^−1^ of 1. Altogether, the lower PI derivative production yields are more likely due to the inconsistent production performance of the *ΔpglAΔsnaE1* mutant strain, rather than that the acceptance of the halogenated derivatives is limiting.

**Table 1:** List of tested mutasynthons with indication if pristinamycin I derivatives with expected masses have been detected by LC/MS analysis and antibacterial activity observed in bioassays (n.t. = not tested). + = derivative/bioactivity observed; − = not observed

Mutasynthon	Calculated mass of PI derivative	New PI-derivative/mass detected	Bioactivity against
			*B. subtilis*
d-Phg	866.4	+	+
2,5-Dihydro-d-Phg	868.4	+	n.t.
4-Fluoro-l-Phg	884.4	+	+
2-Fluoro-dl-Phg	884.4	+	n.t.
4-Chloro-dl-Phg	900.4	+	+
2-Chloro-dl-Phg	900.4	—	n.t.
4-Bromo-dl-Phg	944.3	—	n.t.
4-Amino-dl-Phg	881.4	—	n.t.
4-Hydroxy-dl-Phg	882.4	+	n.t.
2,2-Di-Phg	942.4	—	n.t.
3-(Trifluoromethyl)-dl-Phg	934.4	—	n.t.
4-(Trifluoromethyl)-l-Phg	934.4	—	n.t.
4-Hydroxy-dl-2-fluoro-Phg	882.4	—	n.t.

**Table 2:** NMR (^1^H 700 MHz, ^13^C 175 MHz) for pristinamycin I (DMSO-d_6_) (1), 6-chloropristinamycin I (CH_3_OH-d_4_) (3) and 6-fluoropristinamycin I (DMSO-d6) (4)

Unit	Position	Pristinamycin I (1)		6-Fluoropristinamycin I (4)		6-Chloropristinamycin I (3)	
		^13^C, mult.	^1^H, mult.	^13^C, mult.	^1^H, mult.	^13^C, mult.^d^	^1^H, mult.
Phg	1	168.5, C		168.6, C		169.6, C	
	2	56.8, CH	5.63, d (8.9)	55.3, CH	5.63, m	57.0, CH	5.63, s
	2-NH		8.48, d (8.9)		8.52, br s		
	3	136.2, C		132.7, C		135.9, C	
	4/8	127.7, CH	6.56, m	130.0, CH^a^	7.24, m	130.6, CH	7.24, br d (8.4)
	5/7	128.7, CH	7.35, m	115.6, CH^b^	7.20, m	129.9, CH	7.37, br d (8.4)
	6	128.3, C	7.34, m	161.8, C^c^		135.2, C	

Opp	9	168.4, C		168.8, C		170.1, C	
	10	56.1, CH	5.21, br d (5.4)	58.6, CH	5.45, m	58.0, CH	5.29, br s
	11	40.7, CH_2_	2.04, m	40.7, CH_2_	2.08, m	41.9, CH_2_	2.25, m
			0.52, dd (15.0, 5.7)		0.57, dd (15.5, 4.0)		0.61, m
	12	203.1, C		203.7, C		206.6, C	
	13	38.5, CH_2_	2.23, ddd (16.3, 11.9, 7.4)	38.6, CH_2_	2.21, m	39.6, CH_2_	2.33, m
			2.14, br d (16.3)		2.12, br d (16.3)		2.24, m
	14	36.0, CH_2_	4.54, br dd (13.6, 7.4)	36.1, CH_2_	4.53, m	37.7, CH_2_	4.70, m
			2.63, ddd (13.6, 11.9, 4.5)		2.63, m		2.76, m

Mdp	15	170.5, C		170.7, C		173.5, C	
	16	53.7, CH	4.99, dd (11.6, 4.5)	53.7, CH	5.03, br s	56.0, CH	5.17, m
	17	35.1, CH_2_	3.28, t (12.1)	35.1, CH_2_	3.23, m	35.1, CH_2_	3.26, m
			2.83, m		2.84, m		3.05, dd (12.5, 4.3)
	18	123.0, C		123.1, C		124.5, C	
	19/23	130.2, CH	6.99, br d (8.6)	130.2, CH	6.99, br d (8.4)	131.5, CH	7.07, br d (8.4)
	20/22	112.9, CH	6.78, br d (8.6)	112.9, CH	6.74, br d (8.4)	114.3, CH	6.75, br d (8.4)
	21	150.4, C		150.3, C		152.0, C	
	24/25	40.3, CH_3_	2.83, s	40.4, CH_3_	2.83, s	40.7, CH_3_	2.90, s
	26	30.8, CH_3_	3.18, s	30.8, CH_3_	3.17, br s	31.3, CH_3_	3.25, br s

Pro	27	173.2, C		172.9, C		173.8, C	
	28	56.8, CH	4.64, t (7.4)	56.9, CH	4.64, t (7.4)	58.8, CH	4.63, t (7.4)
	29	27.3, CH_2_	2.04, m	27.4, CH_2_	2.08, m	28.6, CH_2_	2.17, m
			0.80, m		0.88, m		1.10, m
	30	24.4, CH_2_	1.52, m	24.4, CH_2_	1.56, m	25.5, CH_2_	1.43, m
			1.23, m		1.20, m		
	31	47.3, CH_2_	3.68, m	47.3, CH_2_	3.65, m	49.1, CH_2_	3.68, m
			3.31, m		3.28, m		

Abu	32	168.8, C		168.9, C		171.4, C	
	33	50.8, CH	4.77, dt (9.3, 7.7)	50.8, CH	4.73, q (7.5)	52.8, CH	4.81, m
	33-NH		8.28, d (9.3)		n.o.		
	34	23.5, CH_2_	1.68, dtq (13.2, 7.7, 7.3)	23.7, CH_2_	1.67, dtq (13.2, 7.5, 7.3)	25.3, CH_2_	1.74, m
			1.53, m		1.52, m		1.63, m
	35	10.1, CH_3_	0.79, t (7.3)	10.0, CH_3_	0.79, t (7.3)	10.1, CH_3_	0.92, t (7.4)

Thr	36	166.8, C		n.o.		169.4, C	
	37	54.4, CH	4.91, dd (10.5, 1.1)	54.7, CH	4.9, d (10.3)	56.8, CH	4.99, br s
	37-NH		8.07, d (10.5)		8.52, br s		
	38	71.6, CH	5.66, qd (6.5, 1.1)	71.7, CH	5.63, m	73.2, CH	5.80, qd (6.4, 1.7)
	39	15.9, CH_3_	1.11, d (6.5)	16.0, CH_3_	1.10, d (6.5)	16.4, CH_3_	1.28, d (6.4)

Hpc	40	169.0, C		n.o.		171.0, C	
	41	131.6, C		n.o.		132.1, C	
	42	139.3, CH	7.80, m	n.o.		141.2, CH	7.98, br s
	43	129.5, CH	7.78, m	128.2, CH	7.30, m	130.3, CH	7.63, dd (8.1, 4.4)
	44	126.5, CH	7.58, dd (8.0, 1.7)	126.5, CH	7.23, m	127.5, CH	7.48, d (8.4)
	45	157.5, C		n.o.		n.o.	
	45-OH		11.97, s		11.97, br s		

a Doublet with *J*_C,F_ = 8.6 Hz.

b Doublet with *J*_C,F_ = 21.5 Hz.

c Doublet with *J*_C,F_ = 245 Hz.

d Signals assigned from HSQC and HMBC correlations.

### 2.4 Pristinamycin I mutasynthesis products are antimicrobially active

**Fig. 5:**
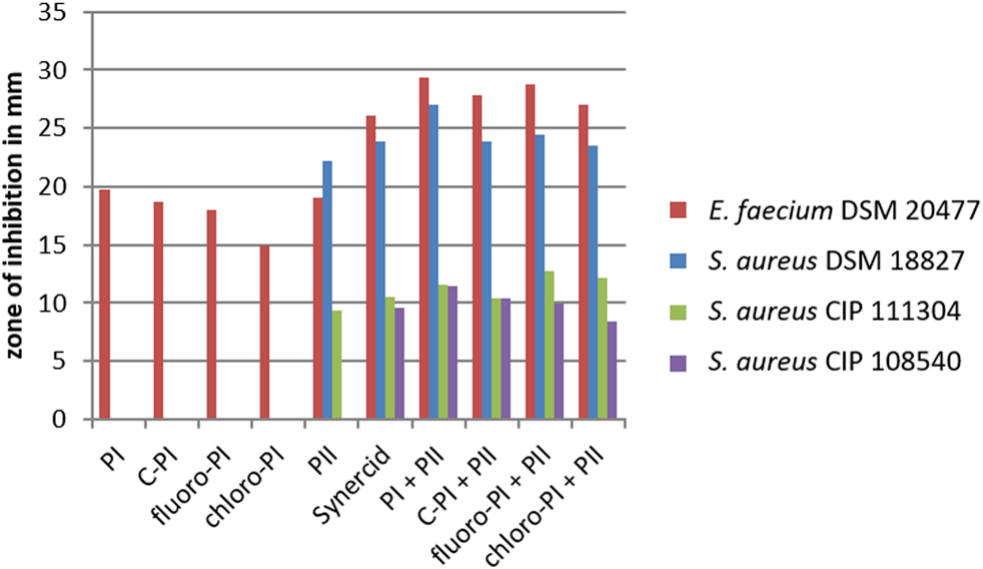
Diameter of inhibition zones caused by pristinamycin I derivatives. Crude extract samples of C-PI, 6-fluoropristinamycin I (6-fluoro-PI), 6-chloropristinamycin I (6-chloro-PI) were used as well as pure pristinamycin I (PI) and II (PII). All S_B_ samples were applied with and without the addition of pristinamycin II. Synercid® filter discs were used as a positive control.

To test, whether the generated pristinamycin I derivatives are antimicrobially active, bioassays were carried out with crude extracts mutasynthesis samples containing 3 and 4. The antibiotic activity of crude extract samples was tested and confirmed against *B. subtilis* (data not shown). Bioactive samples were additionally tested against a panel of clinically relevant microorganisms from the WHO priority list of pathogens, including different Gram-positive and Gram-negative bacteria, as well as fungi. Namely, these were *Staphylococcus aureus* DSM 18827, CIP 111304, and CIP 108540, *Enterococcus faecium* DSM 20477, *Pseudomonas aeruginosa* DSM 1117, *Escherichia coli* DSM 1103, *Proteus vulgaris* DSM 2140, *Candida albicans* DSM 1386, and *Trichophyton rubrum* DSM 16111 (Table S1, ESI†). Of the three tested *S. aureus* strains, two strains (CIP 111304 and CIP 108540) were specifically selected due to their genetically encoded resistance to S_A_ and S_B_ antibiotics. CIP 111304 harbors the genes *vat(A)* and *vgb(A)*, each conferring resistance against S_A_ and S_B_ antibiotics, respectively, whereas CIP 108540 contained the S_A_ resistance genes *vga(A)*, *vga(B)*, and *vat(B)*, as well as the S_B_ resistance genes *erm(A)* and *erm(B)*.^47^ DSM18827 was selected as a multi-resistant test strain^48^ (Table S1, ESI†). Of the different test strains, *Enterococcus faecium* was inhibited by the crude extract samples containing 3 and 4, respectively, to a similar extent as observed with pure pristinamycin I substance and Synercid®, which were used as positive controls (Fig. S22A, ESI†). Bioassays with 1, 3 and 4 were also carried out in combination with pure pristinamycin II compound (10 μg per compound and filter disc). Here, *E. faecium* and the *S. aureus* test strains were inhibited by the respective pristinamycin I derivative plus pristinamycin II combination to a similar extent as with the respective positive control samples, which were pure pristinamycin I + II mixture and Synercid®, respectively (Fig. 5 and Fig. S22A–D, ESI†). Altogether, this showed that 3 and 4 have antimicrobial activities similar to that of the natural compound 1. Furthermore, MIC assays and cytotoxicity tests were performed with pure 4 as this compound was available in sufficient amounts (approx. 1 mg of the pure compound) to allow for additional bioactivity tests. MIC analysis was performed using a panel of standard test organisms, including yeast, fungi, Gram-negative bacteria, as well as *B. subtilis*, *S. aureus*, and *Mycobacterium smegmatis* as Gram-positive test bacteria. Here, the derivative 4 inhibited the growth of *S. aureus* at a concentration of 33.2 μg mL^−1^ and that of *B. subtilis* at a concentration of 4.2 μg mL^−1^, which were 16.7 μg mL^−1^ and 2.1 μg mL^−1^ for the pristinamycin I control, respectively (Table S2, ESI†). Since the MIC values of 4 and 1 resided in a similar range, the generated derivative 4 is obviously similarly bioactive as the natural compound 1. Cytotoxicity assays were carried out with 4 and 1 against two standard human cell lines (KB3.1 and L929), respectively. As with the pristinamycin I (1) control, no effect on proliferation was observed for 4 (Table S3, ESI†). Thus, altogether the 4 derivative shows an equal bioactivity pattern as the natural pristinamycin I (1). So far, this represents the only example of successful mutasynthesis of streptogramin antibiotics.

In a recent study from Walker and Clardy, 2021^49^ on a machine learning bioinformatics approach to predict bioactivities from BGCs, it has been found that BGCs containing Phg encoding genes are explicitly associated with antibacterial activities. Unlike proteinogenic amino acids, Phg does not have a β-carbon, so it has fewer rotatable bonds than proteinogenic aromatic amino acids like *e.g.* phenylalanine. Therefore, Phg-containing peptides should be more rigid, reducing the entropic cost for binding to a target.^50^ Thus overall, we predict to be Phgs a good target residue for derivatization to improve compound properties.

### 2.5 Target related studies with pristinamycin I derivatives

To investigate the inhibitory effect of the pristinamycin I derivatives on protein biosynthesis as an antibiotic target, *in vitro* transcription/translation assays were performed using semi-purified pristinamycin I derivatives 3 and 4 and respective control samples (pristinamycin I (1) and II). Assays were carried out and evaluated as reported before.^51^ Based on previous studies, *iv*TT fluorescence values of 0–20% indicate strong inhibition of the assay, 0–40% are considered to represent specific inhibition, whereas values above 40% do not indicate inhibition of the *iv*TT assay. In *iv*TT assays with pristinamycin derivatives, it was found that pristinamycin II resulted in strong inhibition of the *iv*TT assay, whereas neither pristinamycin I (1) nor the halogenated pristinamycin I derivatives 3 and 4 led to an inhibition (Fig. S23, ESI†). This might be explained by the insufficiency of the *iv*TT assay to sense streptogramin B-mediated protein synthesis inhibition as it has been reported previously that translational inhibition was also not detected in cell-free assays for streptogramin B antibiotics, such as virginiamycin S, quinupristin (2) or pristinamycin I (1).^52–54^

### 2.6 Extended mutasynthesis with *E. coli*-derived mutasynthons

To generally extent the possibility of accessing further, potentially not commercially available Phg derivatives, we established an *E. coli* BL21(DE3) whole cell biotransformation route from halogenated phenylalanines as precursors to produce Phg derivatives (Fig. S3, ESI†). We have demonstrated in a previous study that *E. coli* BL21(DE3) is a suitable microorganism to transform l-phenylalanine derivatives to the corresponding mandelic acid by using a heterologously expressed hydroxymandelate synthase gene *hmaS* from *A. mediterranei*.^55^ To expand the production platform to Phg, we further expressed the genes for mandelate oxidase (*hmo*) from *S. coelicolor* and leucine dehydrogenase (*bcd*) from *B. thuringensis* in a second *E. coli* BL21(DE3) strain. To overcome a NADH cofactor limitation, the glucose dehydrogenase (*gdh*) was additionally expressed in *E. coli* BL21(DE3). To test the substrate spectrum of the Hmo-LeuDH-GDH cascade, a variety of halogenated mandelic acid derivatives were tested. Fluorinated Phgs were detected in the supernatant with a high enantiomeric excess of more than 95%, while chlorinated Phgs were detected with a lower enantiomeric excess of 50% for 2-chloro-l-Phg (Table S4, ESI†).

Mutasynthesis is usually carried out with chemically pure mutasynthons as building blocks that are supplied to the respective biosynthesis mutant. In order to establish a completely fermentative biosynthetic route from the production of the mutasynthon to the final mutasynthesis end product, we developed a mixed mutasynthesis set-up, involving samples from the *E. coli* biotransformation approach that were fed to *S. pristinaespiralis ΔpglAΔsnaE1* as pristinamycin I mutasynthesis strain. This should allow for a direct incorporation of non-commercially available mutasynthons and in principle also provide a more sustainable production route for drug derivatization. To test for the feasibility of such an approach, 100 mL of *S. pristinaespiralis ΔpglAΔsnaE1* main cultures were supplemented with 1 mL of sterile filtered culture supernatants of *E. coli* BL21(DE3) pET28-*hmo*/pACYC-*bcd-gdh*, which contained approx. 1 mg mL^−1^ of 4-fluoro-Phg as representative Phg mutasynthon for pristinamycin I derivatization. LC-MS analysis of crude extracts from *ΔpglAΔsnaE1* fed with 4-fluoro-Phg biotransformation supernatant samples led to mass signals characteristic of 6-fluoro-PI (Fig. S24, ESI†). Thus, 4-fluoro-Phg from *E. coli* supernatant samples was successfully incorporated into pristinamycin I, which proved the feasibility of the adapted mutasynthesis procedure. Thereby, the pristinamycin derivative production rates were comparable to those when supplemented with commercially available mutasynthons (Fig. S24A–F, ESI†). This shows that the biotransformation-based mutasynthesis approach is comparably efficient to conventional mutasynthesis. The extended version of the mutasynthesis approach including biotransformation-derived mutasynthon samples was termed “biotransformation-coupled mutasynthesis”.

## 3. Conclusions

The streptogramin family of antibiotics represents a rare and underexplored family of antibiotics with pristinamycin I (1) as a representative substance in clinical application as an antibiotic of last resort against multi-drug resistant bacterial infections. Substance derivatization is difficult to implement chemically due to the structural complex nature structure of the streptogramin molecule(s). So far, the mutasynthesis of streptogramin antibiotics has been prevented by the lack of knowledge on precursor biosynthesis and the encoded genes. In this study, we present the first successful mutasynthesis approach for the derivatization of the streptogramin antibiotic pristinamycin I (1) based on a modification of the Phg residue. Two halogenated pristinamycin I derivatives, 6-chloropristinamycin I (3) and 6-fluoropristinamycin I (4), were generated by mutasynthesis, their chemical structures were confirmed by NMR and the bioactivity of both substances was demonstrated with antibacterial bioassays. The pristinamycin I mutasynthesis approach was extended by a biotransformation process using a genetically modified *E. coli* strain as a Phg mutasynthon producer, which provided Phg derivatives that were directly fed to the mutasynthesis strain. This mutasynthesis 2.0 process represents a novel extension of the original mutasynthesis method and the feasibility of the process has been demonstrated by the successful production of 46-fluoropristinamycin I (4).

## 4. Material and methods

### 4.1 Bacterial strains and plasmids

The bacterial strains and plasmids used in this study are listed in Table S1 (ESI†). For routine cloning work, *Escherichia coli* Novablue (Novagen) (Sigma-Aldrich, Germany) was used. *S. pristinaespiralis* PR11 (Sanofi-Aventis) was used for pristinamycin production analysis and *S. pristinaespiralis* M*pglA*^41^ served as the host strain for the generation of the *S. pristinaespiralis* mutasynthesis strain. The biological samples used for the experiments were obtained from the Leibniz Institut DSMZ – Deutsche Sammlung von Mikroorganismen und Zellkulturen GmbH, Braunschweig, Germany.

### 4.2 Media and cultivation conditions

Strain cultivation procedures for *S. pristinaespiralis* were carried out as described before.^56^ For cultivation and harvesting of genomic DNA, *S. pristinaespiralis* strains were grown in 100 mL of S-medium^57^ in 500 mL Erlenmeyer flasks (with steel springs) on an orbital shaker (180 rpm) at 28 °C. Kanamycin (50 μg mL^−1^), apramycin (50 μg mL^−1^), and thiostrepton (50 μg mL^−1^) were used for selection when appropriate. For pristinamycin production analysis, *S. pristinaespiralis* strains were grown in 100 mL of the HT7T medium.^58^

### 4.3 Preparation and manipulation of DNA

Total DNA isolation from *S. pristinaespiralis* was performed as described by Kieser *et al.*, 2000^57^ and with the DNA Nucleospin Microbial DNA kit (Bioanalysis, Macherey-Nagel, Germany), respectively. Plasmid isolation was performed using the peqGold Plasmid MiniPrep kit (VWR, Life Science, USA), Pure Yield Plasmid MidiPrep System kit (Promega, USA), or according to Sambrook *et al.*, 1989.^59^ PCR products were purified from 1% agarose gel using the Wizard® SV Gel and PCR Clean-Up System kit (Promega, USA). Enzymes, including restriction endonucleases, ligase, and Q5 DNA polymerase were used according to the manufacturer's recommendations (New England Biolabs, USA; Thermo Fischer Scientific, USA). The primers used for PCR were obtained from MWG Biotech AG (MWG; Ebersberg, Germany) and are listed in Table S1 (ESI†). The primers used for PCR were obtained from MWG Biotech AG (MWG; Ebersberg, Germany) and are listed in Table S1 (ESI†).

### 4.4 Cloning procedure for obtaining mutasynthesis strain *S. pristinaespiralis ΔpglAΔsnaE1*

The mutasynthesis strain *S. pristinaespiralis ΔpglAΔsnaE1* was generated using the following procedure: A 2.4 kb fragment (*snaE1*′), covering an internal part of the *snaE1* gene, was amplified with PCR using genomic DNA from strain *S. pristinaespiralis* PR11 as template and primer pair MsnaE1fw/rv (Table S1, ESI†). The *snaE1*′ amplificate was subcloned into the *Eco*RV-restricted *E. coli* vector pDRIVE, resulting in the construct pDRIVE/snaE1′. The *snaE1*′ fragment was isolated from pDRIVE/snaE1′ as an *Eco*RI fragment and then ligated into the *Eco*RI-restricted *E. coli* vector pK18, resulting in the construct pK18/snaE1′. A 1.1 kb thiostrepton resistance cassette (*tsr*^*R*^) was isolated as a *Sna*BI/*Ale*I fragment from pDRIVE/thio and cloned into the *Stu*I restriction site of *snaE1*′, resulting in the mutational construct pK18/snaE1tsr (Fig. S25, ESI†). The plasmid was transferred to the *pglA* deletion mutant *S. pristinaespiralis* M*pglA*^41^ by protoplast transformation, followed by selection for apramycin/thiostrepton-resistant and kanamycin-sensitive transformants, which resulted in the double mutant *S. pristinaespiralis ΔpglAΔsnaE1*. The correctness of the mutant was verified by PCR using genomic DNA from *ΔpglAΔsnaE1*. For verification of the *snaE1* gene inactivation, primer pairs were used, which annealed to the *tsr*^R^ cassette (thio1/2) and to internal parts of the *snaE1* gene (K*snaE1*fw/rv), respectively. A 0.4 kb fragment was amplified with the primer pair thio1/2, a 2.1 kb fragment with primer pair K*snaE1*fw/rv, a 0.8 kb fragment with the primer pair K*snaE1*fw/thio2, and a 1.7 kb fragment with the primer pair thio1/K*snaE1*rv, confirming the correctness of the *S. pristinaespiralis ΔpglAΔsnaE1* mutant (Fig. S26, ESI†).

### 4.5 Mutasynthesis with Phg derivatives

For mutasynthesis experiments with *S. pristinaespiralis*, strains were grown in 100 mL of HT7T medium as a preculture. After 72 hours, 10 mL of preculture was used to inoculate 100 mL of fresh HT7T medium as the main culture. For feeding experiments, the main cultures were supplemented with different mutasynthons (Table 1) (solubilized with 70 μL of 1 N NaOH and neutralized with 50 μL of 1 N HCl per mg compound) to reach a final concentration of 10 μg mL^−1^. The cells were grown for 168 hours at 28 °C under shaking at 180 rpm. To perform mutasynthesis with *E. coli* biotransformation samples, main cultures were prepared as described above and supplemented with 1 mL of *E. coli* BL21(DE3) pET28-*hmo*/pACYC-*bcd-gdh* culture supernatant containing approx. 1 mg mL^−1^ of the respective mutasynthon.

### 4.6 Compound extraction and purification

For initial compound detection and bioassay testing with *S. pristinaespiralis* samples, 5 mL of culture was extracted with 5 mL of ethyl acetate (1 : 1) for 1 h at room temperature (RT) in an overhead shaker. After centrifugation at 5000 rpm for 20 min, ethyl acetate phases were concentrated *in vacuo* completely and then dissolved in 500 μL of methanol. Crude extracts were used for bioassays, high-performance liquid chromatography (HPLC) and high-performance liquid chromatography-mass spectrometry (HPLC-MS, HPLC-MS/MS) analysis. Batch cultures of *S. pristinaespiralis ΔpglAΔsnaE1* fed with chlorinated or fluorinated mutasynthons were grown, extracted, and treated for compound purification following Protocol S1 and S2, respectively. In total, 12 l and 6 l of culture were extracted for 3 and 4 isolation, respectively. Pure fractions were used to elucidate the structure of halogenated derivatives using NMR.

### 4.7 HPLC and HPLC-ESI-MS(/MS) analyses of pristinamycin variants

HPLC analyses of pristinamycin derivatives were performed using an HP1090M system with ChemStation 3D software rev. A.08.03 (Agilent Technologies, Waldbronn, Germany) on a Waters Symmetry C18 (3.0 × 150 mm, 5 μm) with a flow rate of 850 μL min^−1^. Chromatography was done by linear step gradient elution from 80% solvent A (water with 0.1% phosphoric acid) and 20% solvent B (acetonitrile to 50% solvent B over 12 min, 50% solvent B to 100% solvent B over 3 min followed by a 3 min hold of 100% solvent B). The injection volume was 5 μL. Multiple wavelength monitoring was performed at 210, 230, 260, 280, 310, 360, 435 and 500 nm. UV-Vis spectra were measured from 200 to 600 nm. The evaluation of the chromatograms (210 nm only) was done by means of an in-house HPLC-UV-Vis database. HPLC-MS and HPLC-MS/MS analyses of pristinamycin derivatives were performed using an Agilent 1200 series chromatography system (binary pump, high performance autosampler, DAD-detector) coupled with an LC/MSD Ultra Trap System XCT 6330 (Agilent Technologies, Waldbronn, Germany). The sample (2.5 μL) was injected on a Nucleosil 100 C18 column (100 × 2 mm, 3 μm) fitted with a precolumn (10 × 2 mm, 3 μm) at a flow rate of 400 μL min^−1^ and a linear gradient from 90% solvent A (0.1% formic acid in water) to 100% solvent B (0.06% formic acid in acetonitrile) over 15 minutes at 40 °C. UV-Vis-detection was done at 220, 260, 280, 360 and 435 nm. Electrospray ionization was performed in positive and negative ultra-scan modes (alternating) with a capillary voltage of 3.5 kV and a drying gas temperature of 350 °C. Detection of *m*/*z* values was conducted with Agilent DataAnalysis for 6300 Series IonTrap LC/MS Version 3.4 (Bruker Daltonik). Upon HPLC-MS analysis, pristinamycin I derivatives were identified by comparison of their UV-Vis spectra, retention times and molecular masses with an authentic pristinamycin I (PI) standard (Sanofi-Aventis) and predicted masses for 1 = *m*/*z* 867.4034 [M + H]^+^, and derivatives 3 = *m*/*z* 901.3646 [M + H]^+^, and 4 = *m*/*z* 885.3941 [M + H]^+^.

Pristinamycin I (1): white powder; 
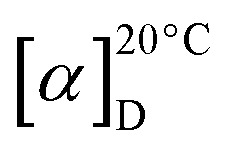
 −50 (*c* 0.6 g/100 mL, MeOH); UV (MeOH) *λ*_max_ (log *ε*) 202 (4.8), 260 (4.2), 304 (3.9); ESI-MS: *m*/*z* 865.32 [M−H]^−^ and 867.56 [M + H]^+^; HR-ESI-MS: *m*/*z* 867.4034 [M + H]^+^ (calculated for sum formula C_45_H_55_N_8_O_10_, 867.4036).

6-Chloro-PI (3): white to slightly brown powder; UV (MeOH) *λ*_max_ (log *ε*) 201 (4.9), 255 (4.3), 302 (4.0); ^1^H NMR data (700 MHz, CH_3_OH-d_4_): see Table 2; ^13^C NMR data (175 MHz, CH_3_OH-d_4_): see Table 2; ESI-MS: *m*/*z* 899.47 [M−H]^−^ and 901.44 [M + H]^+^; HR-ESI-MS: *m*/*z* 901.3645 [M + H]^+^ (calculated for C_45_H_54_N_8_O_10_Cl, 901.3646).

6-Fluoro-PI (4): white powder; 
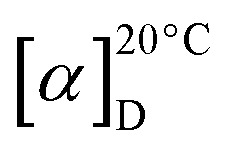
 −45 (*c* 0.1 g mL^−1^, MeOH); UV (MeOH) *λ*_max_ (log *ε*) 203 (4.8), 260 (4.2), 303 (3.9); ESI-MS: *m*/*z* 883.55 [M−H]^−^ and 885.54 [M + H]^+^; ^1^H NMR data (700 MHz, DMSO-d_6_): see Table 2; ^13^C NMR data (175 MHz, DMSO-d_6_): see Table 2; ^19^F NMR (470 MHz, DMSO-d_6_): *δ*_F_ 113.38 ppm; HR-ESI-MS: *m*/*z* 885.3945 [M + H]^+^ and 907.3762 [M + Na]^+^ (calculated C_45_H_54_N_8_O_10_F, 885.3941 and C_45_H_53_N_8_O_10_FNa, 907.3748).

Atom numbering refers to Oku *et al.* 2004.^60^

### 4.8 Structure elucidation

NMR spectra were recorded using an Avance III 700 spectrometer with a 5 mm TCI cryoprobe (^1^H 700 MHz, ^13^C 175 MHz) and an Avance III 500 spectrometer (^1^H 500 MHz, ^13^C 125 MHz) (both Bruker BioSpin, Rheinstetten, Germany). Chemical shifts *δ* were referenced to DMSO-d_6_ (*δ*_H_ = 2.50 ppm; ^13^C, *δ*_C_ = 39.51 ppm), CHCl_3_-d (*δ*_H_ = 7.27 ppm; ^13^C, *δ*_C_ = 77.0 ppm) and trifluoroacetic acid (*δ*_F_ = −74.95 ppm in DMSO).

ESI mass spectra were recorded with an UltiMate 3000 Series uHPLC (Thermo Fisher Scientific, Waltman, MA/USA) by utilizing a C18 Acquity UPLC BEH column (50 × 2.1 mm, 1.7 μm; Waters, Milford, USA) connected to an amaZon speed ESI-Iontrap-MS (Bruker Daltonics, Bremen, Germany). The HPLC parameters were set as follows: solvent A: H_2_O + 0.1% formic acid, solvent B: acetonitrile (MeCN) + 0.1% formic acid, gradient: 5% B for 0.5 min, increasing to 100% B over 19.5 min, keeping 100% B for a further 10 min, flow rate 0.6 mL min^−1^, and DAD detection 190–600 nm.

HRESIMS was performed using a maXis ESI-TOF (electrospray ionization-time of flight) mass spectrometer (Bruker GmbH, Bremen, Germany) coupled to an Agilent 1260 series HPLC-UV system equipped with a C18 Acquity UPLC BEH 2.1 × 50 mm, 1.7 μm (Waters) column; DAD-UV detection at 200–600 nm; solvent A (H_2_O) and solvent B (MeCN) supplemented with 0.1% FA as a modifier; flowrate of 0.6 mL min^−1^, 40 °C oven temperature, gradient elution system with stationary phase of 0.5 min at 5% B, followed by an increase from 5% to 100% B in 19.5 min and holding at 100% B for 5 min.

### 4.9 Computational docking studies of pristinamycins with the ribosome

For computational docking studies with pristinamycins, SeeSAR version 13.0.1 was used; BioSolveIT GmbH, Sankt Augustin, Germany, 2023, https://www.biosolveit.de/SeeSAR. PDB files of the co-crystal structures of quinupristin (2) with the ribosome of *E. coli* (PDB: 4U26), *D. radiodurans* (PDB: 1MS1), and *H. marismortui* (PBD: 1YJW)^34,35,44^ were downloaded from https://www.rcsb.org/. Fragment growing and truncation approaches were performed based on the structure of quinupristin (2) and poses of pristinamycin I (1) as well as new derivatives with modifications of the Phg moiety were generated and re-docked into the binding pockets of quinupristin (2).

### 4.10 Disk diffusion bioassays

General antibiotic activity of pristinamycin variants was analyzed by disc diffusion assays using *Bacillus subtilis* ATCC 6633 as the test organism and was carried out as reported before.^61^ Additionally, a panel of pathogenic organisms including, Gram-positive and Gram-negative bacteria, as well as eukaryotic organisms was used (Table S1, ESI†). 30 μL of *S. pristinaespiralis* culture extracts were pipetted on a filter disc, which was placed on a *Bacillus subtilis* test plate. Synercid® filter discs (15 μg; Oxoid) were used as a positive control and 30 μL of methanol as a negative control. The plates were incubated overnight at 37 °C. The bioactivity of the samples was determined qualitatively by measuring the diameter of the inhibition zone. For more quantitative activity tests of halogenated pristinamycin I derivatives 3 and 4 using streptogramin susceptible pathogenic test organisms (*Enterococcus faecium* DSM 20477 and *Staphylococcus aureus* (DSM 18827, CIP 111304, CIP 108540)), the amount of used extract was calculated to contain approximately 10 μg of the respective derivative and was added to filter discs with or without 10 μg of pristinamycin II, respectively.

### 4.11 Minimal inhibition concentration (MIC) tests

Compound 4 was tested in MIC assays in serial dilutions to determine the minimum inhibitory concentration against standard test organisms, which included fungi, Gram-positive and Gram-negative test bacteria (Table S2, ESI†). Appropriate antibiotics were used as positive controls. Assays were performed following the protocol described by Becker *et al.*^62^

### 4.12 Cytotoxicity tests

Compound 4 was tested in cytotoxicity assays against two different mammalian cell lines, including human endocervical adenocarcinoma KB 3.1 and mouse fibroblasts L929 cell lines, with epothilone B as a positive control (Table S3, ESI†). Assays were performed following the protocol described by Becker *et al.*^62^

### 4.13 *In vitro* transcription and translation (*iv*TT) assay with pristinamycin I derivatives

Semi-purified HPLC fractions of 1, 3, and 4 were tested in *iv*TT assays. Assays were carried out as reported before^51^ and were conducted in triplicates. Each assay had an end concentration of approximately 0.5 μg mL^−1^ of 1, 3, 4, or PI standard (Sanofi-Aventis) with or without the addition of 0.5 μg mL^−1^ pristinamycin II standard (Sanofi-Aventis), respectively. Fluorescence of generated eGFP was measured using a Varioskan LUX plate reader (Thermo Scientific) to quantify the effect of tested samples on transcription/translation.

### 4.14 Construction of a whole cell biotransformation route for Phg mutasynthon production

In order to biotechnologically produce Phg mutasynthons, a synthetic enzymatic cascade was applied (Fig. S27, ESI†). A whole cell biotransformation route with resting cells was established by heterologous expression of the genes encoding hydroxymandelate synthase (*hms*) from *Amycolatopsis mediterranei*, mandelate oxidase (*hmo*) from *S. coelicolor*, leucine dehydrogenase (*bcd*) from *Bacillus thuringensis*, and glucose dehydrogenase (*gdh*) from *B. megaterium* in *E. coli* BL21(DE3). The construction of the plasmids used is described in Protocol S3. For the formation of cells used for whole cell biotransformation, production of Hms was done as described before.^55^ Coproduction of the Hmo, Bcd and Gdh was performed in 2 L shaking flasks containing 400 mL of LB medium (kanamycin (50 μg mL^−1^) and chloramphenicol (30 μg mL^−1^). The media were inoculated with an overnight culture to an OD_600_ of 0.02 and incubated at 37 °C and 180 rpm to an OD_600_ of 0.8. Expression was induced with 0.1 mM IPTG and further incubated at 30 °C and 100 rpm for 4 h. Cells were harvested by centrifugation (10 min, RT, 4000 × *g*) and washed twice with 200 mM Tris–HCl buffer (pH 7.5). The optical density at 600 nm was monitored photometrically using a Cary 50 Bio spectrophotometer (Varian, Mulgrave, Australia). The biotransformation was performed in two steps in 250 mL shaking flasks with a reaction volume of 20 mL. The transformation of the phenylalanine derivative to the corresponding mandelic acid was performed as described before.^55^ The supernatant was isolated after 24 h of incubation by centrifugation (10 min, RT, 4000 × *g*) and was used directly as a substrate for the transformation to the Phg synthesis with *E. coli* BL21(DE3) pET28-*hmo*/pACYC-*bcd-gdh*. 20 mM glucose and 50 mM ammonium acetate were added after 1 h and 16 h to the HMO-LeuDH-GDH reaction to ensure the cofactor supply. After 24 h of biotransformation, the supernatant was isolated by centrifugation (10 min, RT, 4000 × *g*), sterile filtrated (Filtropur S (0.45 μm), Sarstedt) and stored at −20 °C until use. The respective enantiomeric excess (ee) of the produced Phgs was determined using HPLC analysis with comparison to standards of available racemic and enantiomeric pure Phgs (Fig. S28, ESI†). The separation of Phg enantiomers was achieved by using a Chirex 3126 (d)-penicillamine column (150 × 4.6 mm, Phenomenex, Aschaffenburg, Germany) with 2 mM copper(ii) sulfate and 15% methanol as mobile phase at 30 °C. The isocratic flow was set to 1 mL min^−1^ and 4 μL of sample was injected.

## Author contributions

OH carried out a mutasynthesis experiment with *S. pristinaespiralis*. LW and J-WY performed biotransformation experiments for Phg-derivative supply. KH, FS, and AK performed chemical analysis (HPLC, MS/MS, NMR). KH and FS carried out MHC and cytotoxicity tests. PK carried out SeeSAR modeling analysis. J-WY designed and supervised biotransformation studies. YM designed and supervised mutasynthesis studies and coordinated the study.

## Conflicts of interest

The authors declare that the research was conducted in the absence of any commercial or financial relationships that could be construed as a potential conflict of interest.

## Supplementary Material

CB-004-D3CB00143A-s001

CB-004-D3CB00143A-s002

CB-004-D3CB00143A-s003

CB-004-D3CB00143A-s004
