# Supplementary material for: Biotransformation-coupled mutasynthesis for the generation of novel pristinamycin derivatives by engineering the phenylglycine residue
Source: RSC Chem Biol. 2023 Oct 14;4(12):1050–63. doi: 10.1039/d3cb00143a (PMC10685826; doi:10.1039/d3cb00143a)
Supplement: CB-004-D3CB00143A-s004 [file CB-004-D3CB00143A-s004.pdf]

Electronic Supplementary Material (ESI) for RSC Chemical Biology.
This journal is © The Royal Society of Chemistry 2023

Biotransformation-coupled mutasynthesis for the generation of novel pristinamycin derivatives by engineering the phenylglycine residue

Oliver Hennrich, [a] Leoni Weinmann, [b] Andreas Kulik, [c] Karen Harms, [d] Philipp Klahn, [e,f] Jung-Won Youn, [b] Frank Surup, [d] and Yvonne Mast *[a,g,h]

[a] Department Bioresources for Bioeconomy and Health Research, Leibniz Institute DSMZ - German Collection of Microorganisms and Cell Cultures, Inhoffenstraße 7B, 38124 Braunschweig, Germany

[b] Institute of Microbiology, University Stuttgart, Allmandring 31, D-70569 Stuttgart, Germany

[c] Department Microbial Bioactive Compounds, Interfaculty Institute of Microbiology and Infection Medicine, Faculty of Science, University of Tübingen, Auf der Morgenstelle 28, D-72076 Tübingen, Germany

[d] Microbial Drugs Department, Helmholtz-Centre for Infection Research, 38124 Braunschweig, Germany

[e] Division of Organic and Medicinal Chemistry, Department of Chemistry and Molecular Biology, University of Gothenburg, Kemigården 4, 412 96 Göteborg, Sweden

[f] Centre of Antimicrobial Resistance Research in Gothenburg (CARe)

[g] Technische Universität Braunschweig, Institut für Mikrobiologie, Rebenring 56, 38106 Braunschweig, Germany

[h] German Center for Infection Research (DZIF), Partner Site Tübingen, Tübingen, Germany

*Correspondence:
Prof. Dr. Yvonne Mast
yvonne.mast@dsmz.de

Running title: Mutasynthesis with phenylglycines

Key words: actinomycetes, antibiotic, genetic engineering, mutasynthesis, phenylglycine

Table of Contents

Protocol S1: Fermentation and purification of 6-chloropristinamycin I (**3**)................................4
Protocol S2: Fermentation and purification of 6-fluoropristinamycin I (**4**)................................4
Protocol S3: Construction of plasmids pET28a-*hmo* and pACYC-*bcd-gdh* biotransformation approach for the synthesis of L-Phg derivatives...5
Table S1: Bacterial strains, plasmids and oligonucleotides used in this study............................6
Table S2: MIC assay of pristinamycin IA (**1**) and its 6-fluoro derivative (**4**)................................7
Table S3: Cytotoxicity assay of 6-fluoropristinamycin I (**4**) against human cell lines.................8
Table S4: Determination of enantiomeric excesses (ee) of L-Phg synthons from the whole cell biotransformation approach with *E. coli* BL21(DE3) pET28-*hmo/* pACYC-*bcd-gdh* with different mandelic acids...8
Figure S1: MS/MS fragmentation pattern of **1, 3** and **4**...9
Figure S2: HRESIMS of 6-chloropristinamycin I (**3**)...10
Figure S3: HRESIMS of 6-fluoropristinamycin I (**4**)...11
Figure S4: HRESIMS of pristinamycin I (**1**)...12
Figure S5: ^{1}H NMR spectrum (700 MHz) pristinamycin I (**1**) in DMSO-d_6...................................13
Figure S6: ^{13}C NMR spectrum (700 MHz) pristinamycin I (**1**) in DMSO-d_6.................................14
Figure S7: COSY NMR spectrum (700 MHz) pristinamycin I (**1**) in DMSO-d_6.............................15
Figure S8: TOCSY NMR spectrum (700 MHz) pristinamycin I (**1**) in DMSO-d_6...........................16
Figure S9: ROESY NMR spectrum (700 MHz) pristinamycin I (**1**) in DMSO-d_6...........................17
Figure S10: HSQC-DEPT NMR spectrum (700 MHz) pristinamycin I (**1**) in DMSO-d_6.................18
Figure S11: HMBC NMR spectrum (700 MHz) pristinamycin I (**1**) in DMSO-d_6.........................19
Figure S12: ^{1}H NMR spectrum (700 MHz) 6-chloropristinamycin I (**3**) in MeOD.......................20
Figure S13: COSY NMR spectrum (700 MHz) 6-chloropristinamycin I (**3**) in MeOD..................21
Figure S14: HSQC-DEPT NMR spectrum (700 MHz) 6-chloropristinamycin I (**3**) in MeOD.......22
Figure S15: HMBC NMR spectrum (700 MHz) 6-chloropristinamycin I (**3**) in MeOD................23
Figure S16: ^{1}H NMR spectrum (700 MHz) 6-fluoropristinamycin I (**4**) in DMSO-d_6...................24
Figure S17: ^{13}C NMR spectrum (700 MHz) 6-fluoropristinamycin I (**4**) in DMSO-d_6..................25
Figure S18: HMBC spectrum (700 MHz) 6-fluoropristinamycin I (**4**) in DMSO-d_6......................26
Figure S19: COSY spectrum (700 MHz) 6-fluoropristinamycin I (**4**)in DMSO-d_6.........................27
Figure S20: HSQC-DEPT NMR spectrum (700 MHz) 6-fluoropristinamycin I (**4**) in DMSO-d_6...28
Figure S21: ^{19}F NMR spectrum (470 MHz) 6-fluoropristinamycin I (**4**)in DMSO-d_6...................29
Figure S22: Results of the agar diffusion test of PI derivatives alone and in combination with PII against streptogramin susceptible clinical isolates......................................30
Figure S23: *In vitro* transcription translation assay with of semi purified PI derivatives, as well as pure PI and PII...31
Figure S24: HPLC/MS analysis of *S. pristinaespiralis ΔpglAΔsnaE1* extracts from cultures supplemented with 4-fluoro-Phg containing *E. coli* BL21(DE3) pET28-*hmo*/ pACYC-*bcd-gdh* supernatant...32
Figure S25: Cloning chart of mutagenesis plasmid pK18/snaE1tsr...33
Figure S26: Verification of the *snaE1* mutation by PCR..33

Figure S27: Schematic representation of the biotransformation approach for the synthesis of L-Phg derivatives……………………………………………………………………………………………34
Figure S28: Representative chiral HPLC chromatogram of the separation of phenylglycine enantiomers at 210 nm……………………………………………………………..………………………….34
References………35

Protocol S1: Fermentation and purification of 6-chloropristinamycin I (**3**)

For batch cultivations, 100 mL of HT7T medium was inoculated with 1 mL of mycelium of *S. pristinaespiralis ΔpglAΔsnaE1* and cultivated for 72 h in 500 mL Erlenmeyer flasks (with baffles) on an orbital shaker (110 rpm) at 28°C as a preculture. 100 mL of preculture was used to inoculate 1 L of HT7T medium (supplemented with 100 µM 4-chloro-DL-Phg solubilized as mentioned above) in a 5-L Erlenmeyer flask (with baffles) on an orbital shaker (110 rpm) at 28°C for 48-72h. The whole culture was extracted with two times 1 L ethyl acetate and the organic phase was concentrated in a rotary evaporator. For initial purifications, the extract was first dissolved in 20 mL of methanol/water (ratio: 85/15) and mixed twice with 20 mL of heptane. After separation, the heptane phase was discarded. The mixture was adjusted to a 70/30 methanol/water ratio and mixed twice with 20 mL of dichloromethane. The dichloromethane phase was mixed with 20 mL of water. After discarding the water phase, the extract was evaporated in a rotary evaporator and weighed. In total, 12 L of culture were extracted. For isolation of **3**, 180 mg of the crude extract solved in MeOH were separated using a PLC 2250 preparative HPLC system (Gilson, Middleton, WI, USA) with a Gemini® 10u C18 110Å column (250 × 50 mm, 10 µm; Phenomenex, Torrance, CA, USA) as the stationary phase and in the following conditions: solvent A: H_2O + 0.1% formic acid, solvent B: MeCN + 0.1% formic acid; flow: for 1 min 20 mL/min, afterwards 45 mL/min, fractionation: 15 mL, gradient: increase from 5% B to 100% B in 41 min followed by a final isocratic step of 100% B for 10 min. This yielded one pure fraction of **3** (F3: 0.6 mg, retention time (tR) = 34.02–35.15 min).

Protocol S2: Fermentation and purification of 6-fluoropristinamycin I (**4**)

For batch cultivations, 100 mL of HT7T medium was inoculated with 1 mL of mycelium of *S. pristinaespiralis ΔpglAΔsnaE1* and cultivated for 72h in 500-mL Erlenmeyer flasks (with baffles) on an orbital shaker (110 rpm) at 28°C as a preculture. 100 mL of preculture was used to inoculate 1 L of HT7T medium (supplemented with 100 µM 4-fluoro-L-Phg solubilized as mentioned above) in a 5-L Erlenmeyer flask (with baffles) on an orbital shaker (110 rpm) at 28°C for 48-72 h. The whole culture was extracted with two times 1 L ethyl acetate and the organic phase was concentrated in a rotary evaporator. For initial purifications, the extract was first dissolved in 20 mL of 85/15 methanol/water and mixed twice with 20 mL of heptane. After separation, the heptane phase was discarded. The extract was adjusted to 70/30 methanol/water and mixed twice with 20 mL of dichloromethane. The dichloromethane phase was mixed with 20 mL of water. After discarding the water phase, the extract was evaporated in a rotary evaporator and weighed. In total, 6 L of culture were extracted. 320 mg crude extract were subjected to flash chromatography (Grace Reveleris®, Columbia, MD, USA) (silica cartridge 12 g [FlashPure, Büchi Switzerland, USA], solvent A: DCM, solvent B: DCM/MeOH 95%/5%, solvent C: DCM:MeOH 9/1, solvent D: MeOH), gradient: 100% A for 10 min, increasing to 50% B in 2 min, followed by an isocratic step of 50% B for 2 min, increasing to 100% B in 2 min followed by an isocratic step at 100% B for 2 min, followed by a change from 100% B to 50 % A and 50% C with an increase to 80% C in 2 min, followed by a further increase to 100% C in 2 min, followed by a change to 100% D in 10 min and an isocratic step of 100% D for 10 min. One fraction containing **4** was collected with an amount of 73 mg at the retention time 19.6 min. The yielded fraction from the flash-chromatography was separated using a PLC 2250 preparative HPLC system (Gilson, Middleton, WI, USA) with a Gemini® 10u C18 110Å column (250 × 50 mm, 10 µm; Phenomenex, Torrance, CA, USA) as the stationary phase and

in the following conditions: solvent A: H_2O + 0.1% formic acid, solvent B: MeCN + 0.1% formic acid; flow: 45 mL/min, fractionation: 10 mL, gradient: increase from 5% B to 40% B in 10 min followed by an increase to 42% B in 30 min and a isocratic step in 42% B for 3 min, followed by an increase to 100% B in 8 min and an isocratic step in 100% B for 10 min. This yielded to two pure fractions of **4** (F2: 1.54 mg, tR = 43.36–44.21 min; F2.1 (1.88 mg, tR = 42.23–43.36 min).

Protocol S3: Construction of plasmids pET28a-*hmo* and pACYC-*bcd-gdh* biotransformation approach for the synthesis of L-Phg derivatives

Cloning of pET28a-*hmo*

The synthetic codon optimized *hmo* gene (GeneArt, Regensburg, Germany) from *Streptomyces coelicolor* was cloned into pET28a(+). For this purpose, *hmo* as well as the pET28a plasmid were double-digested with *Nde*I and *Bam*HI. The digested *hmo* gene fragment was ligated into the pET28a plasmid with T4 DNA ligase (NEB). The ligation product was transferred to competent *E. coli* DH5α for plasmid multiplication. The isolated plasmid pET28a-*hmo* was verified by sequencing (Eurofins Genomics, Germany).

Cloning of pACYC-*bcd-gdh*

The gene *bcd* from *Bacillus thuringiensis* was amplified from chromosomal DNA using primers bcd-fw/-rv with KOD Hot Start Polymerase (Novagen). The PCR fragment and pET28a were digested with *Nco*I and *BamH*I and ligated with T4 DNA ligase (NEB). The ligation product was transferred to competent *E. coli* DH5α. The isolated plasmid pET28-*bcd* was verified by sequencing (Eurofins Genomics, Germany). The *Hin*dIII restriction sites in the plasmid were deleted by insertion of point mutations with the primer pairs pET28a-Hind-fw/-rv and bcd-Hind-fw/rv.

The glucose dehydrogenase gene (*gdh*) from *Bacillus megaterium* was amplified from chromosomal DNA using primers gdh-fw/-rv with KOD Hot Start Polymerase (Novagen). The PCR fragment and pET28a were digested with *Nde*I and *Bam*HI and ligated with T4 DNA ligase. The constructed plasmid pET28-*gdh* was verified by sequencing (Eurofins Genomics, Germany). The *Hin*dIII restriction site in the plasmid was deleted by insertion of a point mutation with the primer pET28-Hind-fw/rv. The *bcd* gene was amplified with OP-fw/-rv by using pET28-*bcd* as template with the KOD Hot Start Polymerase (Novagen). The *bcd* fragment was digested with *Kpn*I and *Spe*I. The *gdh* gene was amplified with OP-fw/-rv from the template pET28-*gdh* with the KOD Hot Start Polymerase (Novagen). The *gdh* fragment was digested with *Nhe*I and *Hin*dIII. The *Nhe*I restriction site in pACYC was deleted by insertion of a point mutation with the primer pACYC-Nhe-fw/rv. Afterwards, the pACYC plasmid was amplified with pACYC-fw/-rv with KOD Hot Start Polymerase (Novagen) and afterwards digested with *Kpn*I and *Hin*dIII. All three digested fragments were ligated with T4 DNA ligase to obtain pACYC-*bcd-gdh,* which was transferred to *E. coli* DH5α. The isolated plasmid was verified by sequencing (Eurofins Genomics, Germany).

Table S1: Bacterial strains, plasmids and oligonucleotides used in this study.

**Strains/ plasmids/ oligonucleotides**	**Genotype/phenotype/sequence**	**Source/ reference**
S. pristinaespiralis		
PR11	Pristinamycin producing strain/wild-type, natural isolate of *S. pristinaespiralis* ATCC 25486	Sanofi-Aventis
M*pglA*	gene interruption of *pglA*, $apra^R$, PI non-producing	Mast *et al*., 2011
Δ*pglA*Δ*snaE1*	gene interruption of *pglA*, $apra^R$, PI non-producing<br>gene interruption of *snaE1*, tsr^R, PII non-producing	This study
E. coli		
Novablue	*endA1 hsdR17* (r_{K12}^- m_{K12}^+) *supE44 thi-1 recA1 gyrA96 relA1 lac* F′[*proA*$^+$*B*$^+$ *lacI*^{q}*Z*Δ*M15*::Tn*10*] (Tet^R)	Novagen, SigmaAldrich
DH5α	*fhuA2 lac(del)U169 phoA glnV44 Φ80' lacZ(del)M15 gyrA96 recA1 relA1 endA1 thi-1 hsdR17*	Novagen, Merck Millipore
**Bioassay test strains**		
Bacillus subtilis ATCC 6633	Bioassay test strain	Pelzer *et al*., 1999
Pseudomonas aeruginosa DSM 1117	Bioassay test strain	DSMZ strain collection
Proteus vulgaris DSM 2140	Bioassay test strain	DSMZ strain collection
Enterococcus faecium DSM 20477	Bioassay test strain	DSMZ strain collection
Staphylococcus aureus DSM 18827	Bioassay test strain	DSMZ strain collection
Staphylococcus aureus CIP 111304	Bioassay test strain; *vat(A), vgb(A)*	Collection de L´Institut Pasteur
Staphylococcus aureus CIP 108540	Bioassay test strain; *vgb(A), vgb(B), vat(B), erm(A), erm(B)*	Collection de L´Institut Pasteur
Candida albicans DSM 1386	Bioassay test strain	DSMZ strain collection
Trichophyton rubrum DSM 16111	Bioassay test strain	DSMZ strain collection
**Plasmids**		
pDRIVE	*lacZ'*-complementation system, amp^R, kan^R	Qiagen
pDRIVE/thio	*lacZ'*-complementation system, amp^R, kan^R, tsr^R	Moosmann et al., 2020
pK18	pUC derivative, *aph*II, *lac*Z'-complementation system	Pridmore, 1987
pK18/snaE1tsr	pK18 derivative, *aph*II, tsr^R, *lac*Z'*α*, *snaE1ab*	This study
pET28a	pET28a, kan^R	Novagen
pET28a-*hms*	pET28a derivate with *hms* from *A. mediterranei*, kan^R	Youn *et al.,* 2019
pET28a-*hmo*	pET28a derivate with *hmo* from *S. coelicolor*, kan^R	This study
pET28a-*bcd*	pET28a derivate with *bcd* from *B. thuringiensis*, kan^R	This study
pET28a-*gdh*	pET28a derivate with *gdh* from *B. megaterium*, kan^R	This study
pACYC	pACYC, cm^R	Chang & Cohen 1978
pACYC-*leuDH-gdh*	pACYC derivate, cm^R	This study
**Oligonucleotides**	**5'→3'**	
MsnaE1fw	AGCCGATGCTGTGCACGATG	This study
MsnaE1rv	CCACGTCGTCCTGGAAGAAG	This study
thio1	CGTTGGTGATTGCCGGTCAG	This study

thio2	GGCGATGCCGAATGTCTTGG	This study
KsnaE1fw	TGTCCCACTCGGGTACCAGG	This study
KsnaE1rv	TTCGAGCGGCAGTCCTACTGG	This study
bcd-fw	ATACCATGGGCACATTAGAAATCTTCGAATACTTAGAAAAATATG	This study
bcd-rv	TTCGGATCCTTAGCGACGGCTAATAATATCGTG	This study
pET28-Hind-fw	GAGCTCCGTCGACATGCTTGCGGCCGC	This study
pET28-Hind-rv	GCGGCCGCAAGCATGTCGACGGAGCTC	This study
bcd-Hind-fw	TGAAGAGCGCATTGCTAGCTTGAAAAATTC	This study
bcd-Hind-rv	GAATTTTTCAAGCTAGCAATGCGCTCTTCA	This study
gdh-fw	TTTCATATGATGTATAAAGATTTAGAAGGAAAAGTAGTTGTCATAA CAGG	This study
gdh-rv	TTTGGATCCTTATCCGCGT-CCTGCTTGGAATG	This study
OP-fw	GCAGGTACCGCGGCCGCGCTAGCGATATAGGCGCCAGCAACCGCA C	This study
OP-rv	AGCAAGCTTCTAGCCGGCCGCTACTAGTCAGCAAAAAACCCCTCAA GACCCGTTT	This study
pACYC-Nhe-fw	CAGTATACACTCCGCCAGCGCTGATGTCCGG	This study
pACYC-Nhe-rv	CCGGACATCAGCGCTGGCGGAGTGTATACTG	This study
pACYC-fw	GTCAAGCTTAAGCTGTCAAACATGAGAATTACAAC	This study
pACYC-rv	ATGGGTACCATTTCAGAATATTTGCCAGAACCGT	This study

Table S2: MIC assay of pristinamycin IA (**1**) and its 6-fluoro derivative (**4**) against standard test organisms. Inhibited organisms are highlighted in bold; n.i. = not identified.

		**1**	**4**	
**test organism**	**DSM-Nr.**	**MIC µg/mL**	**MIC µg/mL**	**positive control**
Schizosaccharomyces pombe	70572	n.i.	n.i.	Nystatin
Pichia anomala	6766	n.i.	n.i.	Nystatin
Mucor hiemalis	2656	n.i.	n.i.	Nystatin
Candida albicans	1665	n.i.	n.i.	Nystatin
Rhodotorula glutinis	10134	n.i.	n.i.	Nystatin
Acinetobacter baumannii	30008	n.i.	n.i.	Ciprofloxacin
Escherichia coli	1116	n.i.	n.i.	Oxytetracyclin
Bacillus subtilis	**10**	**2,1**	**4,2**	Oxytetracyclin
Mycobacterium smegmatis	ATCC 700084	n.i.	n.i.	Kanamycin
Staphylococcus aureus	**346**	**16,7**	**33,3**	Oxytetracyclin
Pseudomonas aeruginosa	PA14	n.i.	n.i.	Gentamycin

Chromobacter violaceum	30191	n.i.	n.i.	Oxytetracyclin

Table S3: Cytotoxicity assay of 6-fluoropristinamycin I (**4**) against human cell lines (* = no changed cells, no cytotoxic activity; ** = no changed cells, low inhibition in proliferation)

**Compound**	**KB3.1 cell line**	**Tox-Nr.**	**L929 cell line**	**Tox-Nr.**
**4**	**	**3548**	*	**3549**

Table S4: Determination of enantiomeric excesses (ee) of L-Phg synthons from the whole cell biotransformation approach with *E. coli* BL21(DE3) pET28-*hmo/* pACYC-*bcd-gdh* with different mandelic acids. *The ee of 4-chloro-L-Phg produced by HMO-LeuDH-GDH could not be determined.

**Product**	**Enantiomeric excess of L-phenylglycine derivatives [%]**
L-phenylglycine	>99
2-fluoro-L-phenylglycine	>95
3-fluoro-L-phenylglycine	>99
4-fluoro-L-phenylglycine	>99
2-chloro-L-phenylglycine	50
3-chloro-L-phenylglycine	67
4-chloro-L-phenylglycine	-*

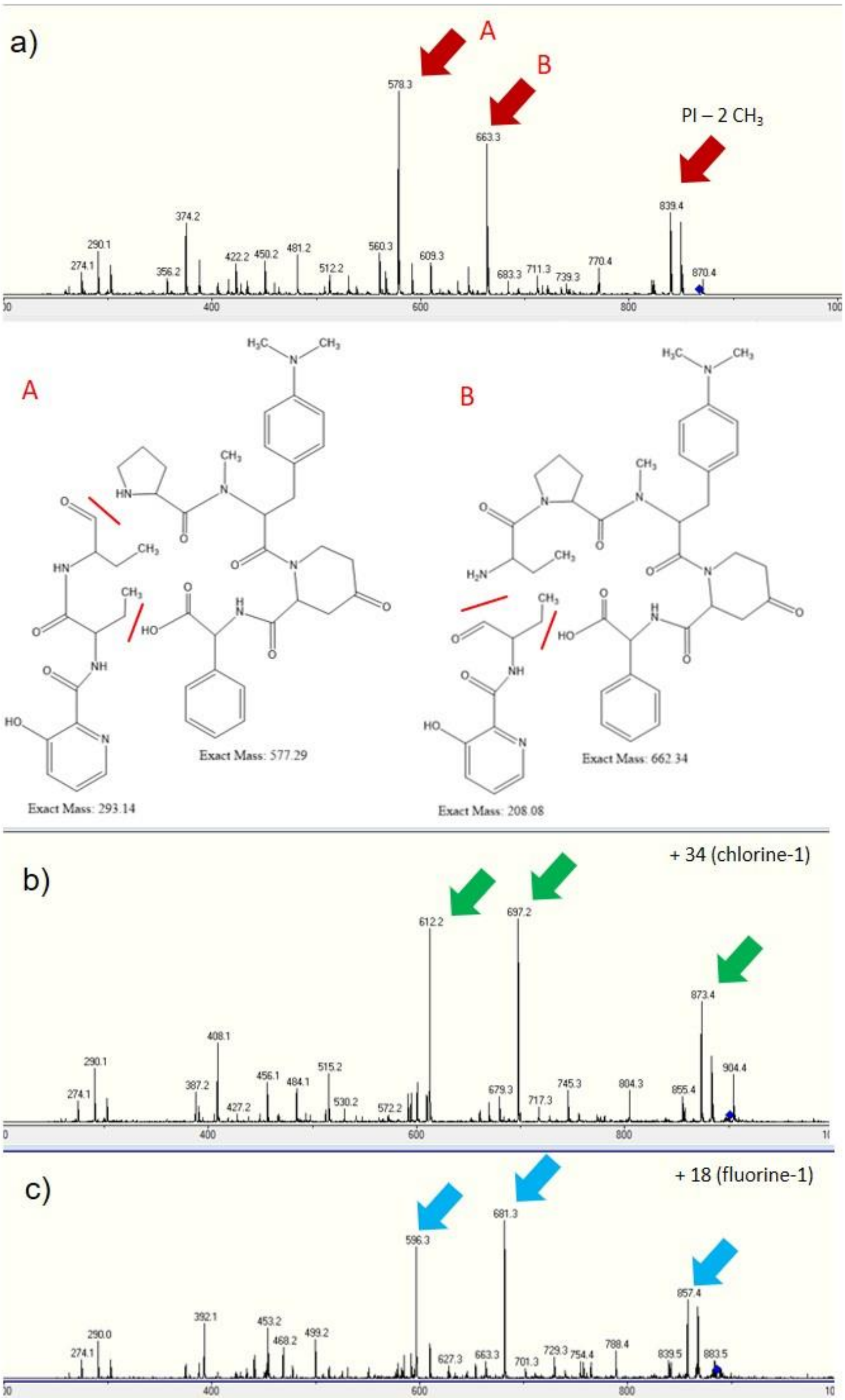

Figure S1: MS/MS fragmentation pattern of pristinamycin I (**1**) (a), 6-chloropristinamycin I (**3**) (b) and 6-fluoropristinamycin I (**4**) (c). Extracts of *S. pristinaespiralis* PR11 (1; m/z of precursor ion: 867.4) and *S. pristinaespiralis ΔpglAΔsnaE1* supplemented with 4-chloro-DL-Phg (2; *m/z* of precursor ion: 901.4) and 4-fluoro-L-Phg (3; *m/z* of precursor ion: 885.4). Distinct fragments of 1 are marked by arrows and likely fragmentation results are shown (A, B). Corresponding fragments that show the expected mass shift for the respective halogenation are marked by arrows in green (chlorination) or blue (fluorination). Measurements were conducted in positive mode.

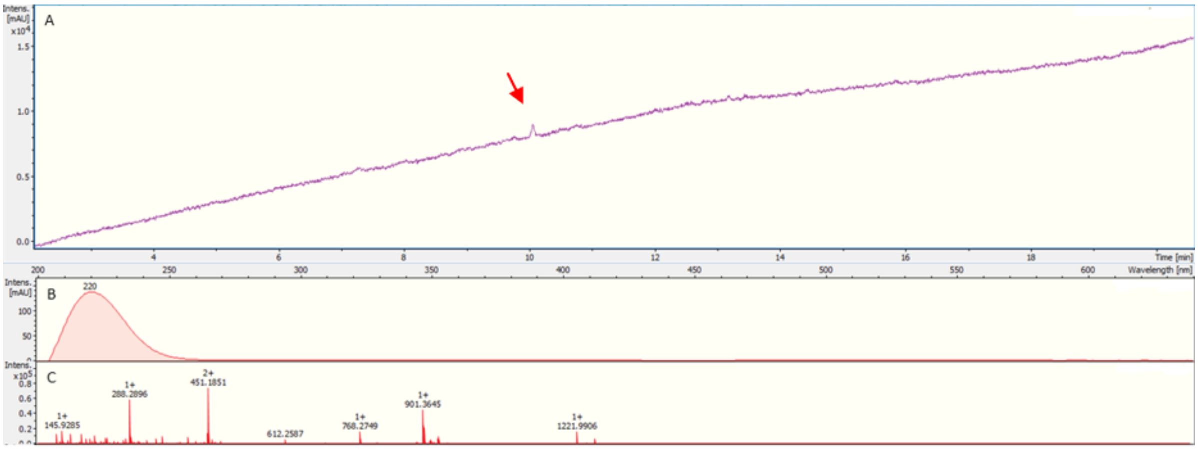

Figure S2: HRESIMS of 6-chloropristinamycin I (**3**). A is the chromatogram at 210 nm (peak indicated by red arrow). B and C are the UV spectrum and the mass spectrum of **3**.

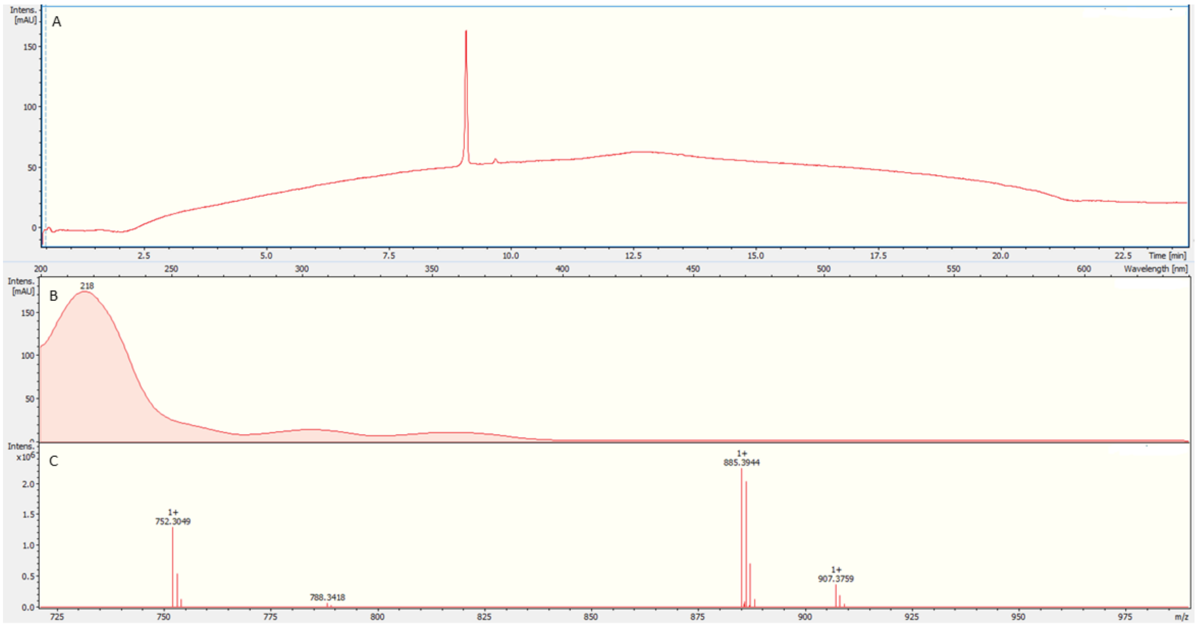

Figure S3: HRESIMS of 6-fluoropristinamycin I (**4**). A is the chromatogram at 210 nm. B and C are the UV spectrum and the mass spectrum of **4**.

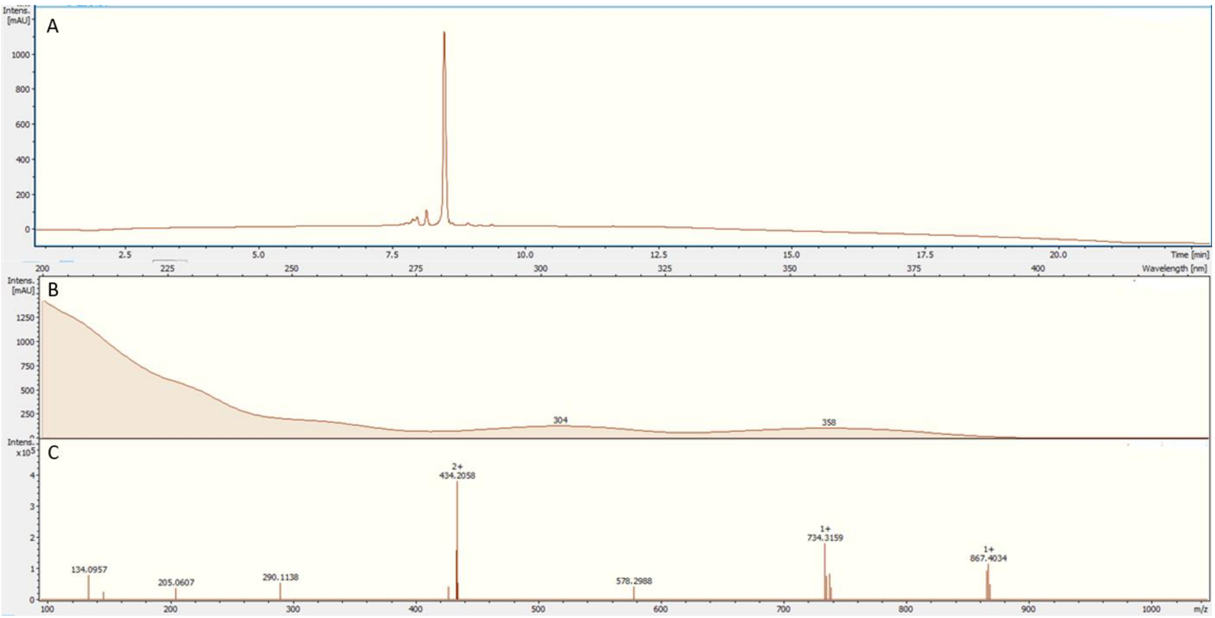

Figure S4: HRESIMS of pristinamycin I (**1**). A is the chromatogram 210 nm. B and C are the UV spectrum and the mass spectrum of **1**.

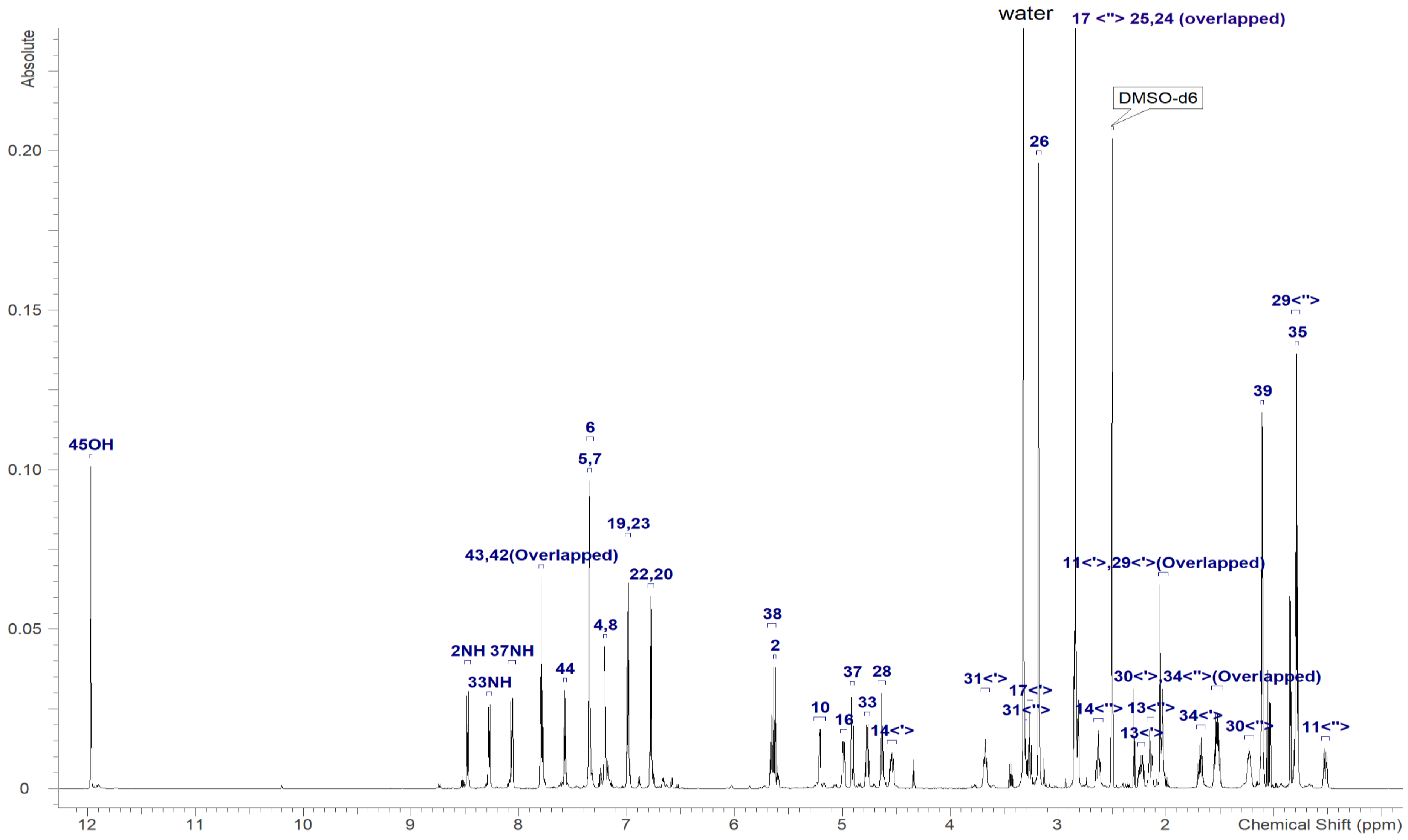

Figure S5: ^{1}H NMR spectrum (700 MHz) pristinamycin I (**1**) in DMSO-d_6.

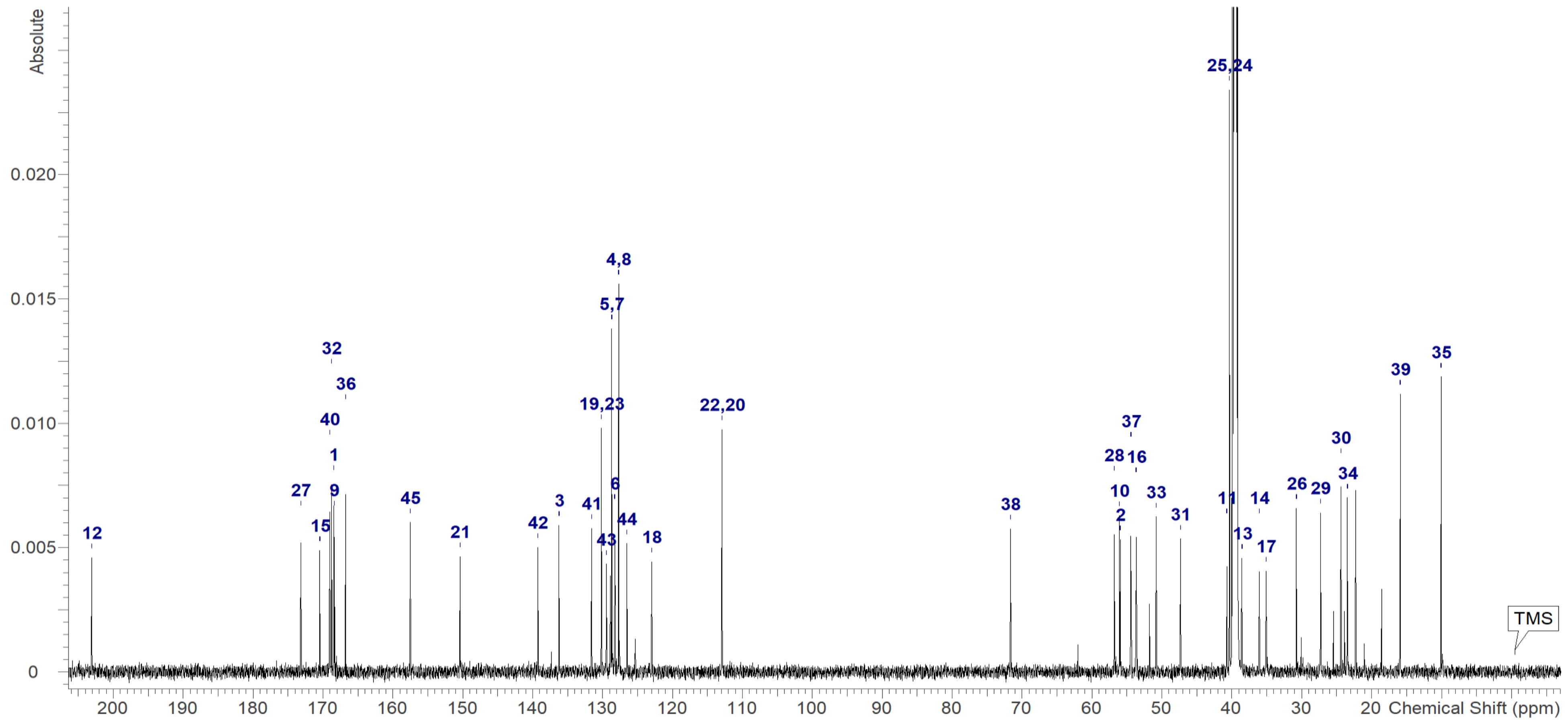

Figure S6: ^{13}C NMR spectrum (700 MHz) pristinamycin I (**1**) in DMSO-d_6.

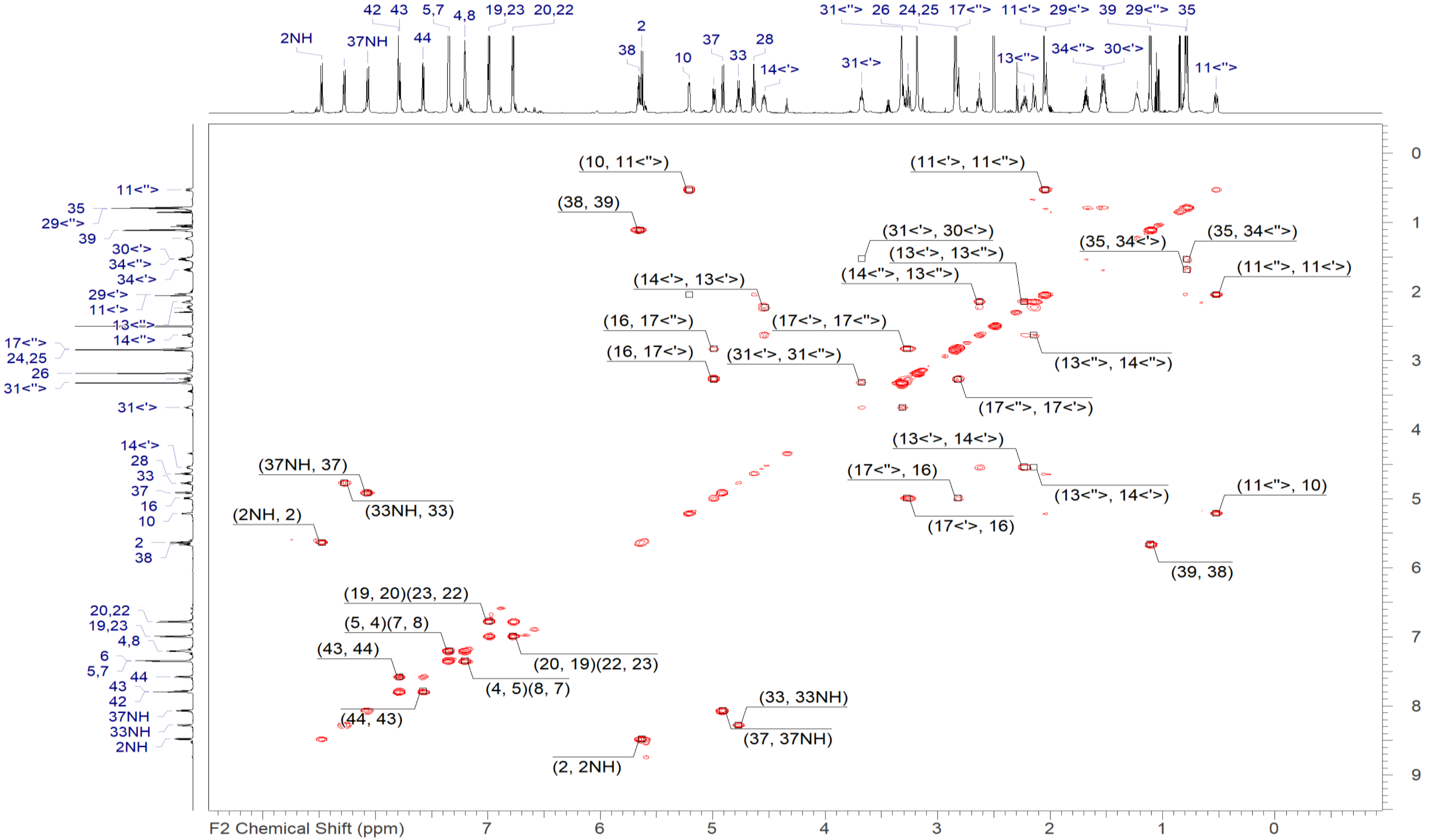

Figure S7: COSY NMR spectrum (700 MHz) pristinamycin I (**1**) in DMSO-d_6.

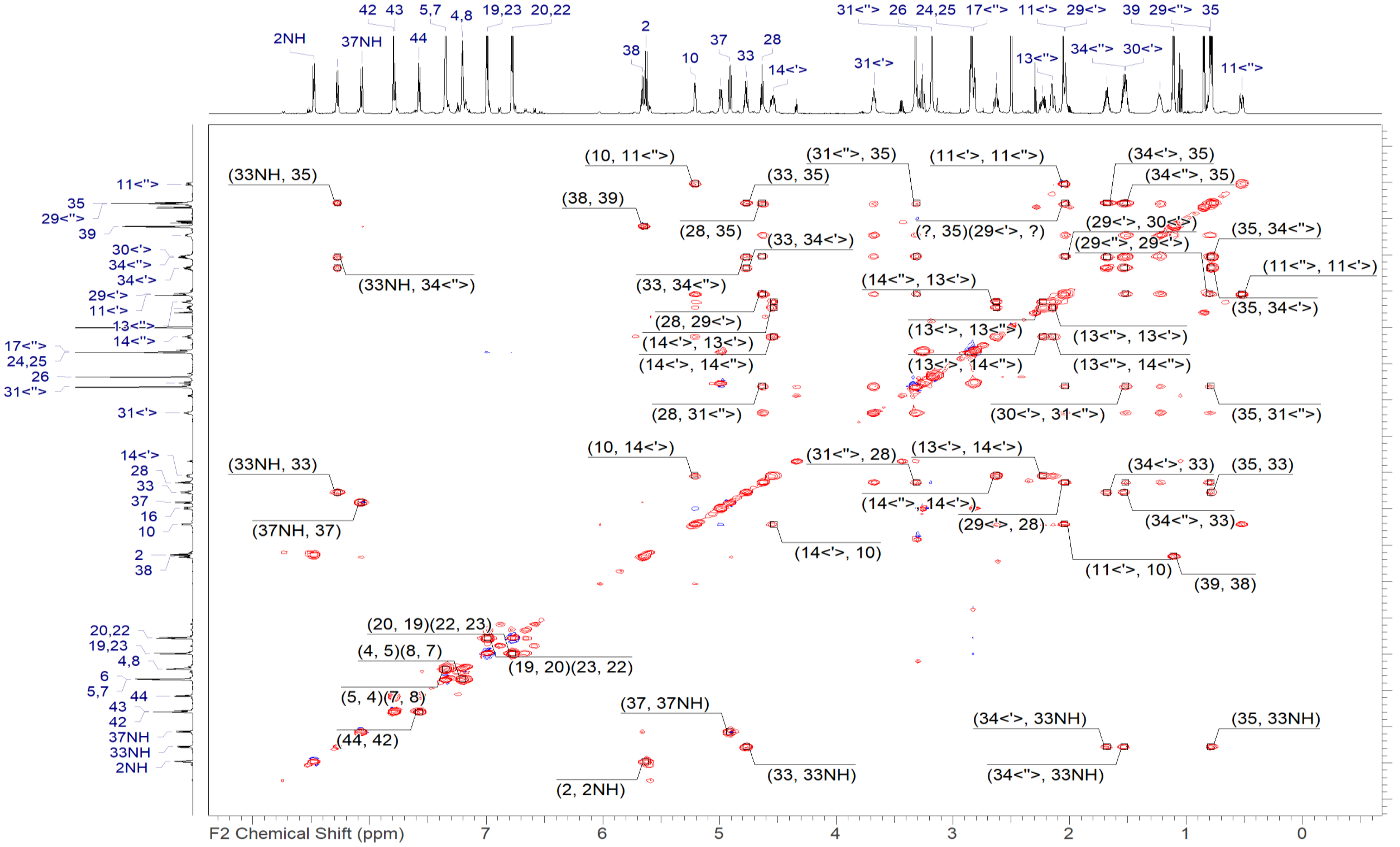

Figure S8: TOCSY NMR spectrum (700 MHz) pristinamycin I (**1**) in DMSO-d_6.

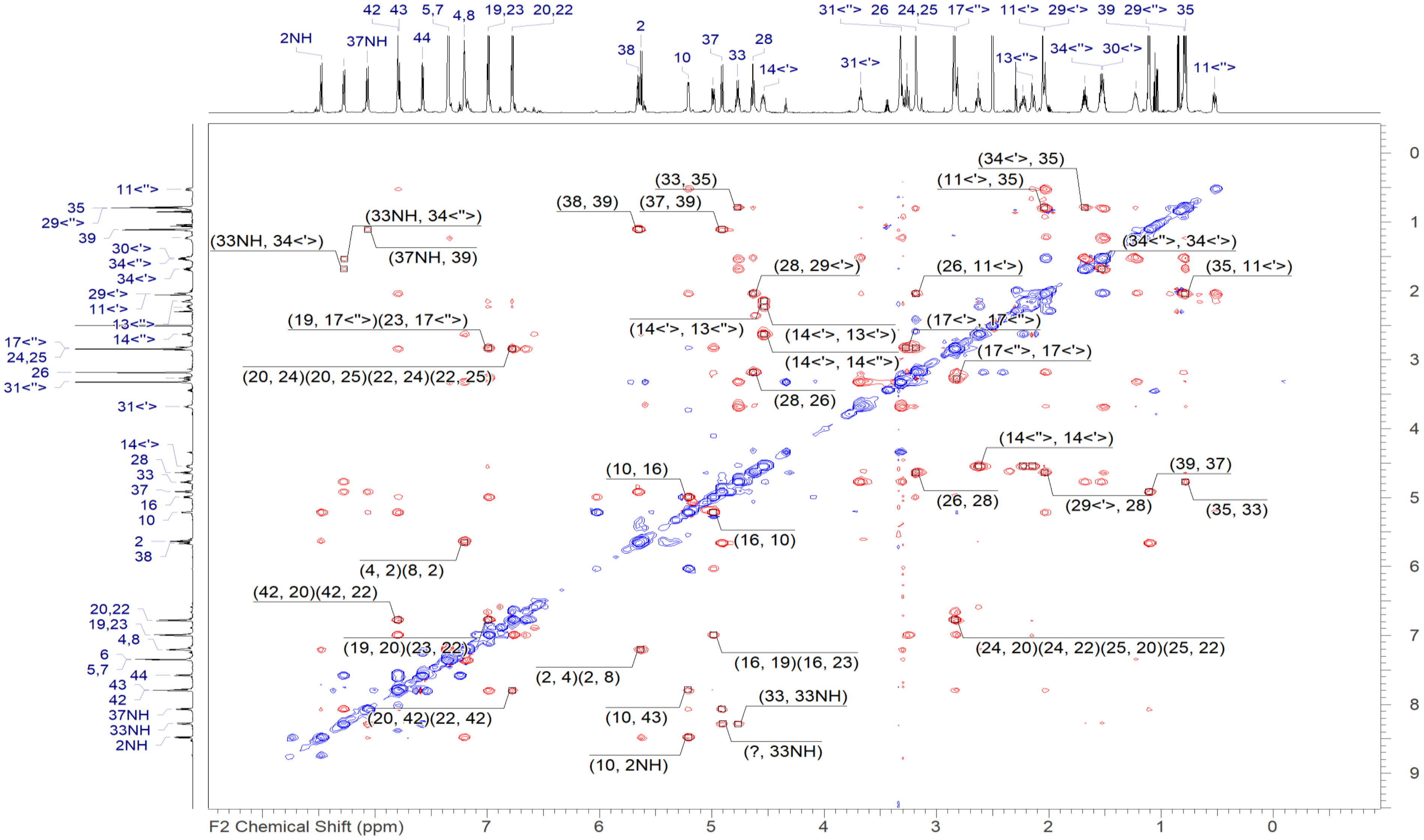

Figure S9: ROESY NMR spectrum (700 MHz) pristinamycin I (**1**) in DMSO-d_6.

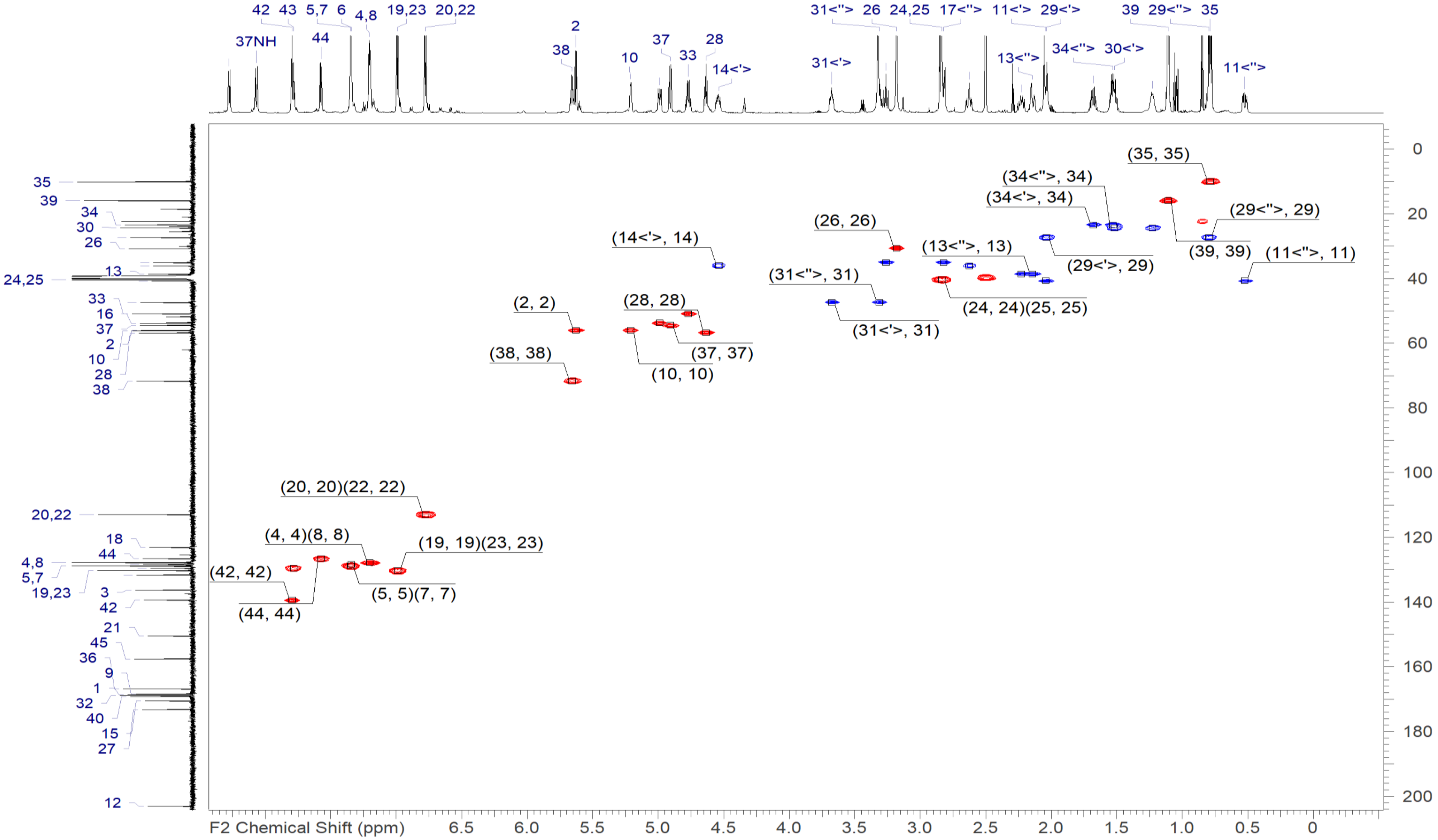

Figure S10: HSQC-DEPT NMR spectrum (700 MHz) pristinamycin I (**1**) in DMSO-d_6.

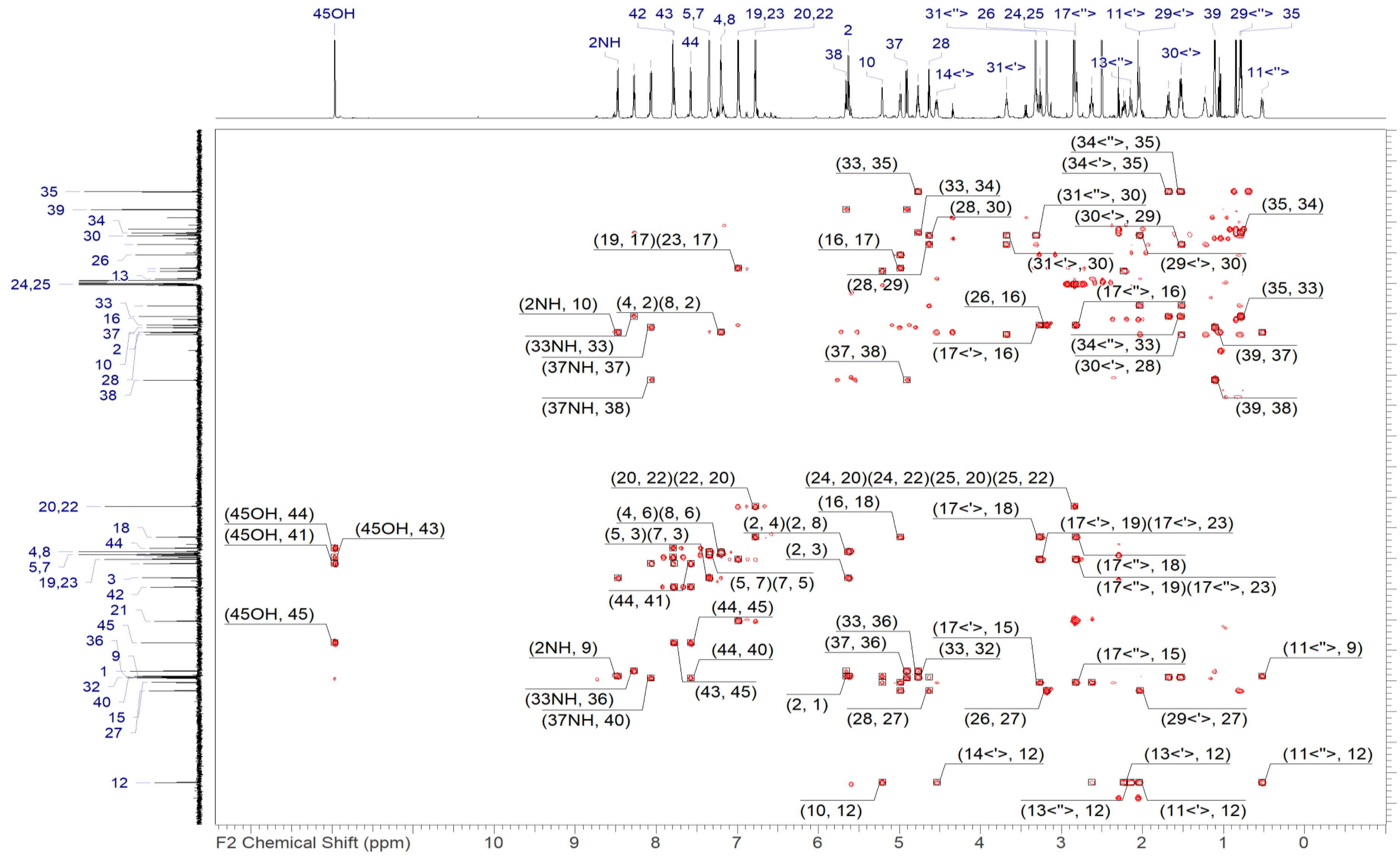

Figure S11: HMBC NMR spectrum (700 MHz) pristinamycin I (**1**) in DMSO-d_6.

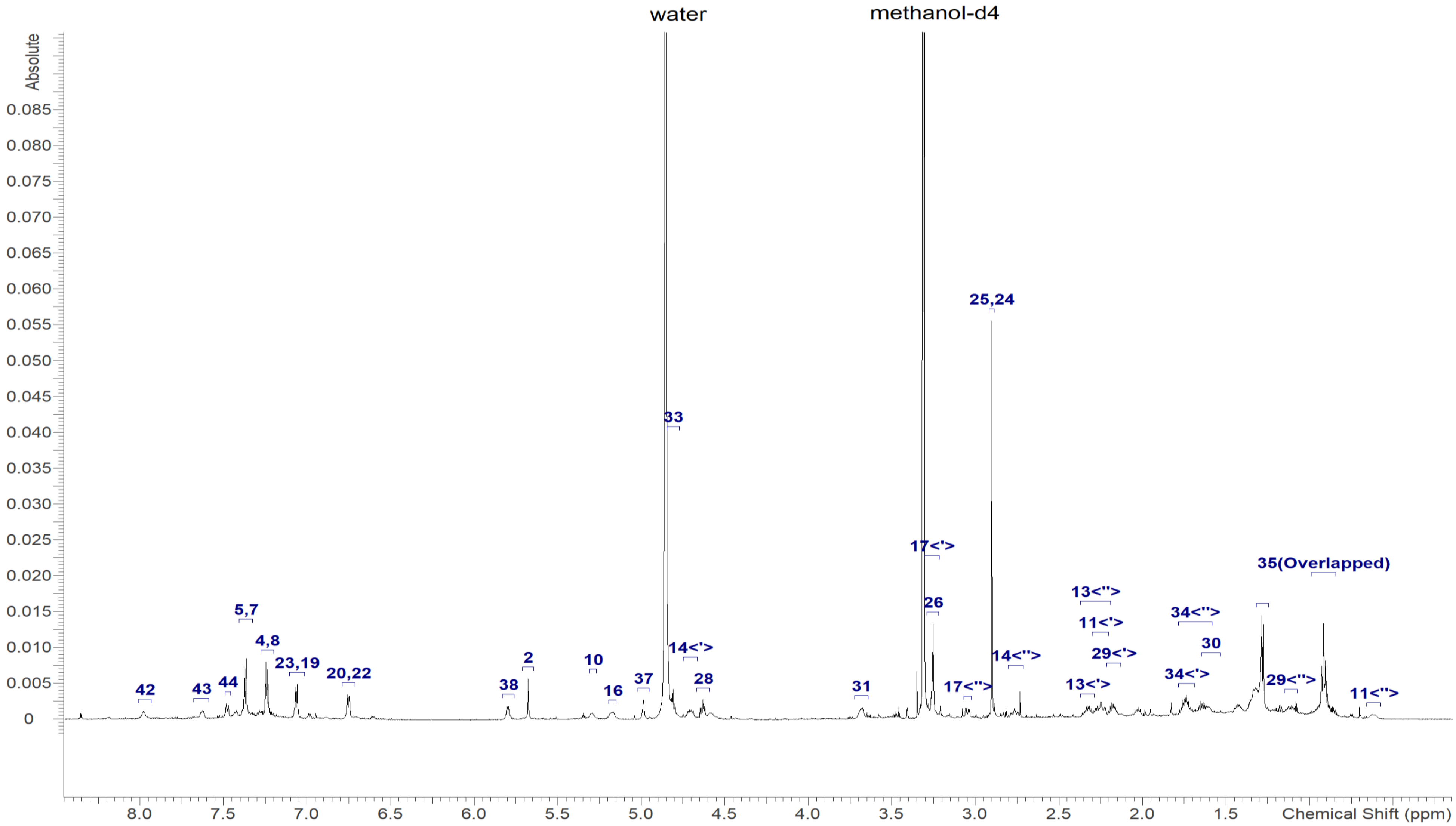

Figure S12: ^{1}H NMR spectrum (700 MHz) 6-chloropristinamycin I (**3**) in MeOD.

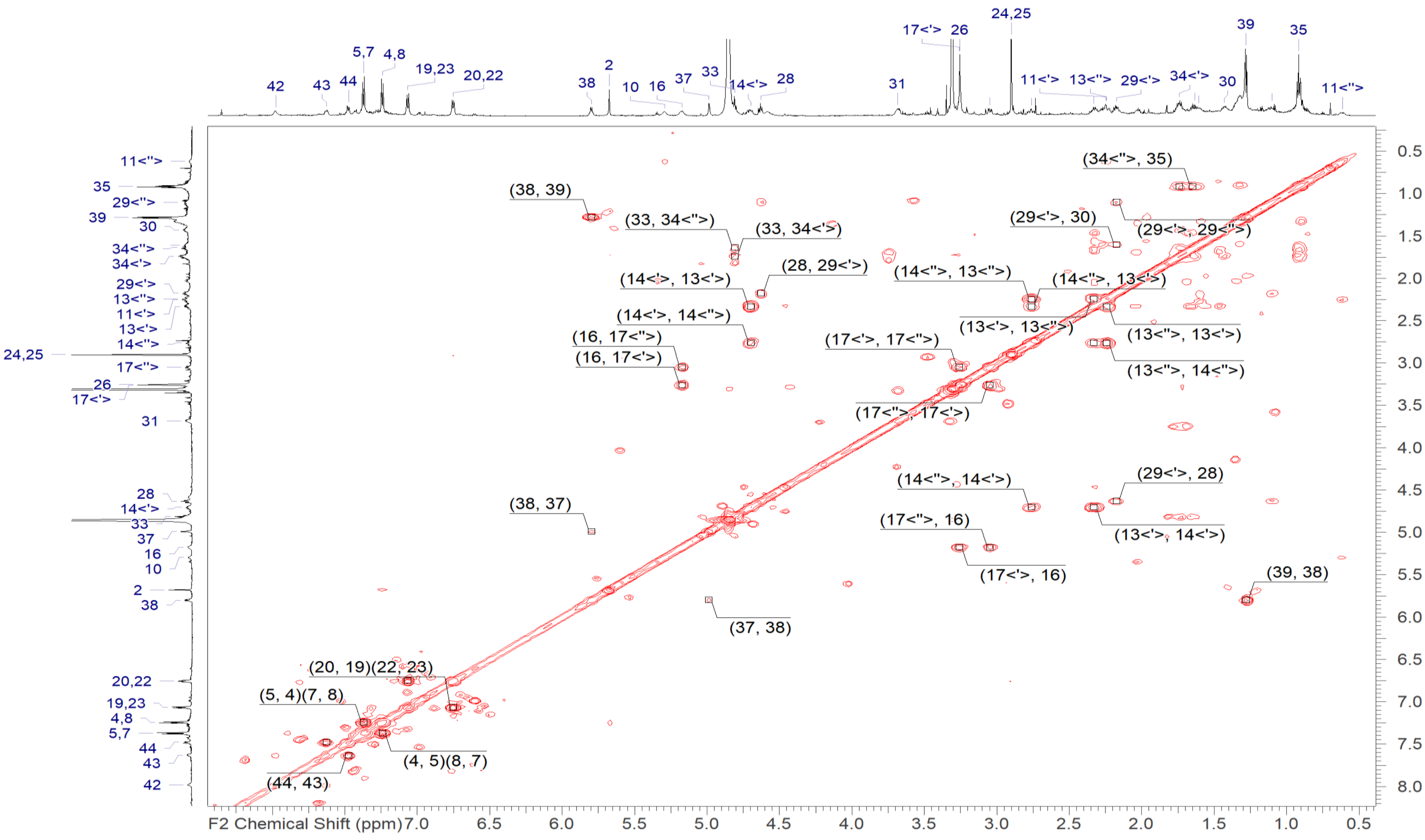

Figure S13: COSY NMR spectrum (700 MHz) 6-chloropristinamycin I (**3**) in MeOD.

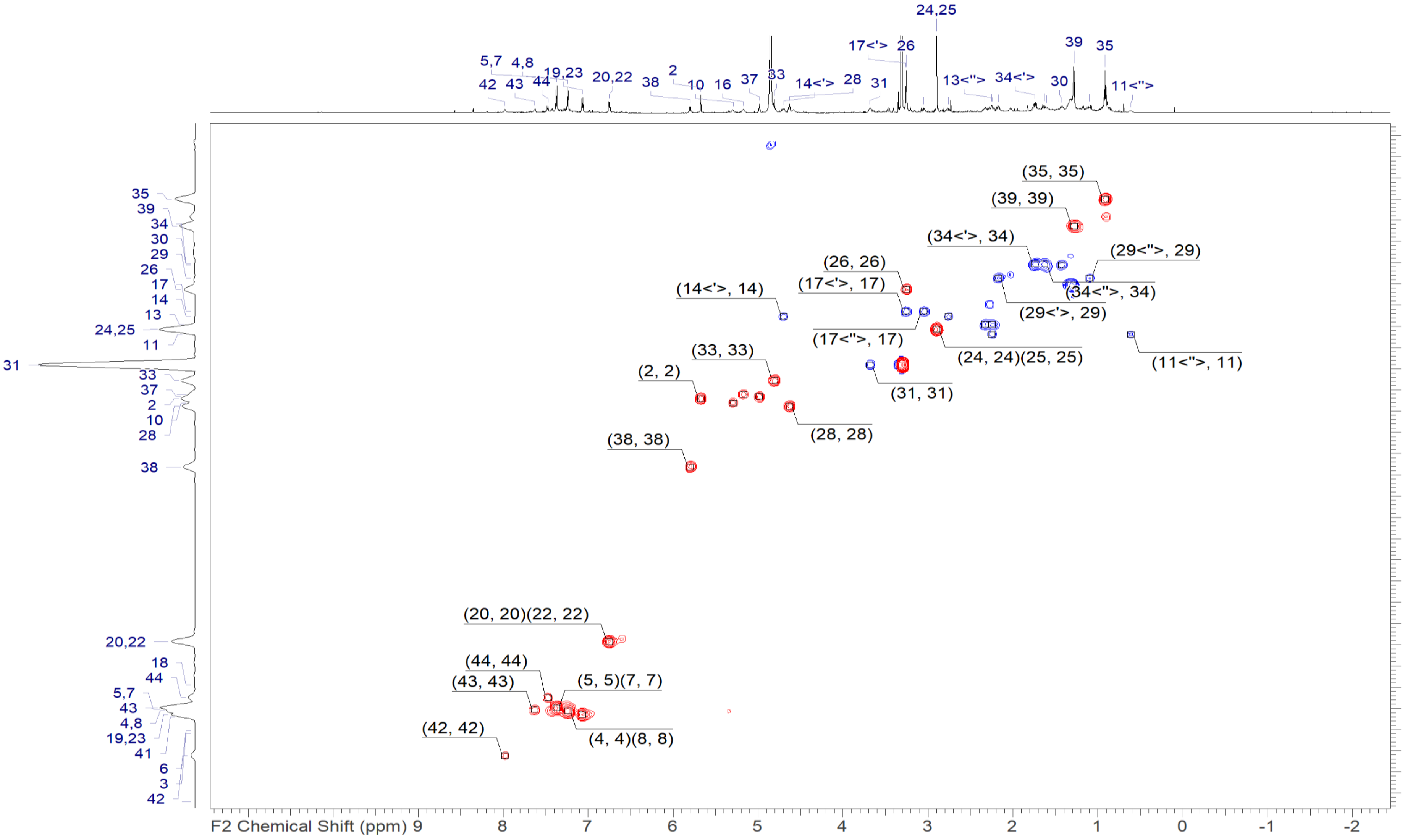

Figure S14: HSQC-DEPT NMR spectrum (700 MHz) 6-chloropristinamycin I (**3**) in MeOD.

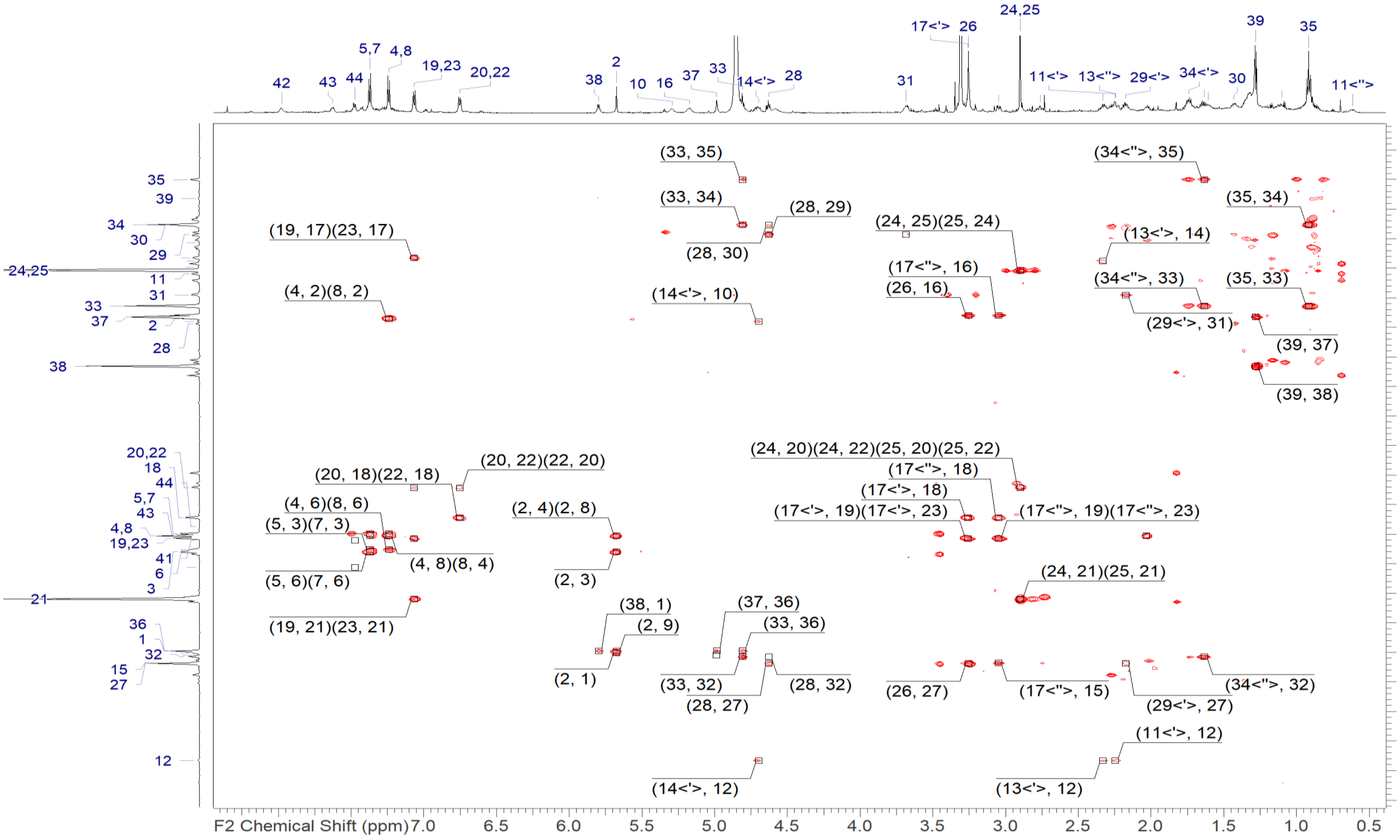

Figure S15: HMBC NMR spectrum (700 MHz) 6-chloropristinamycin I (**3**) in MeOD.

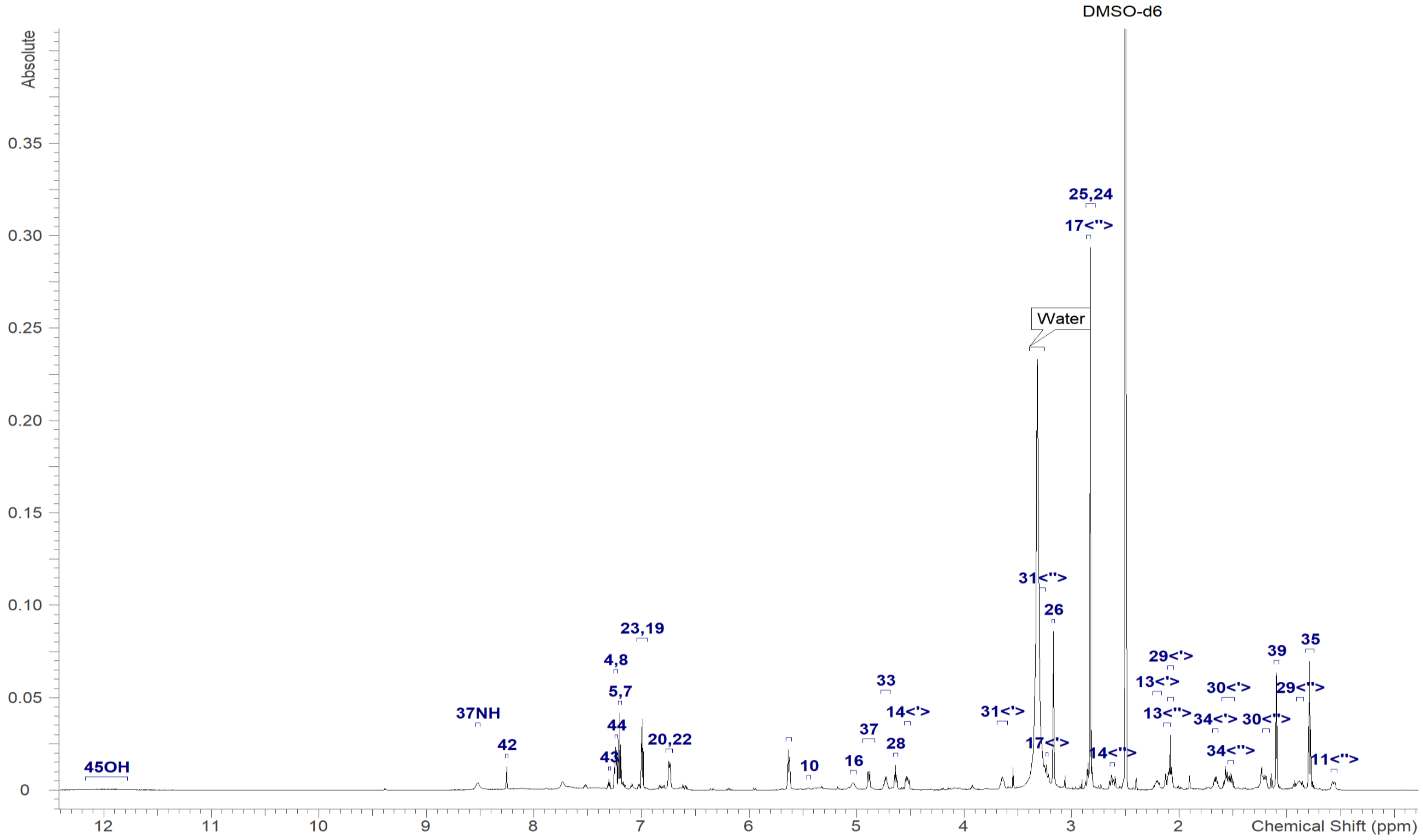

Figure S16 ^{1}H NMR spectrum (700 MHz) 6-fluoropristinamycin I (**4**) in DMSO-d_6.

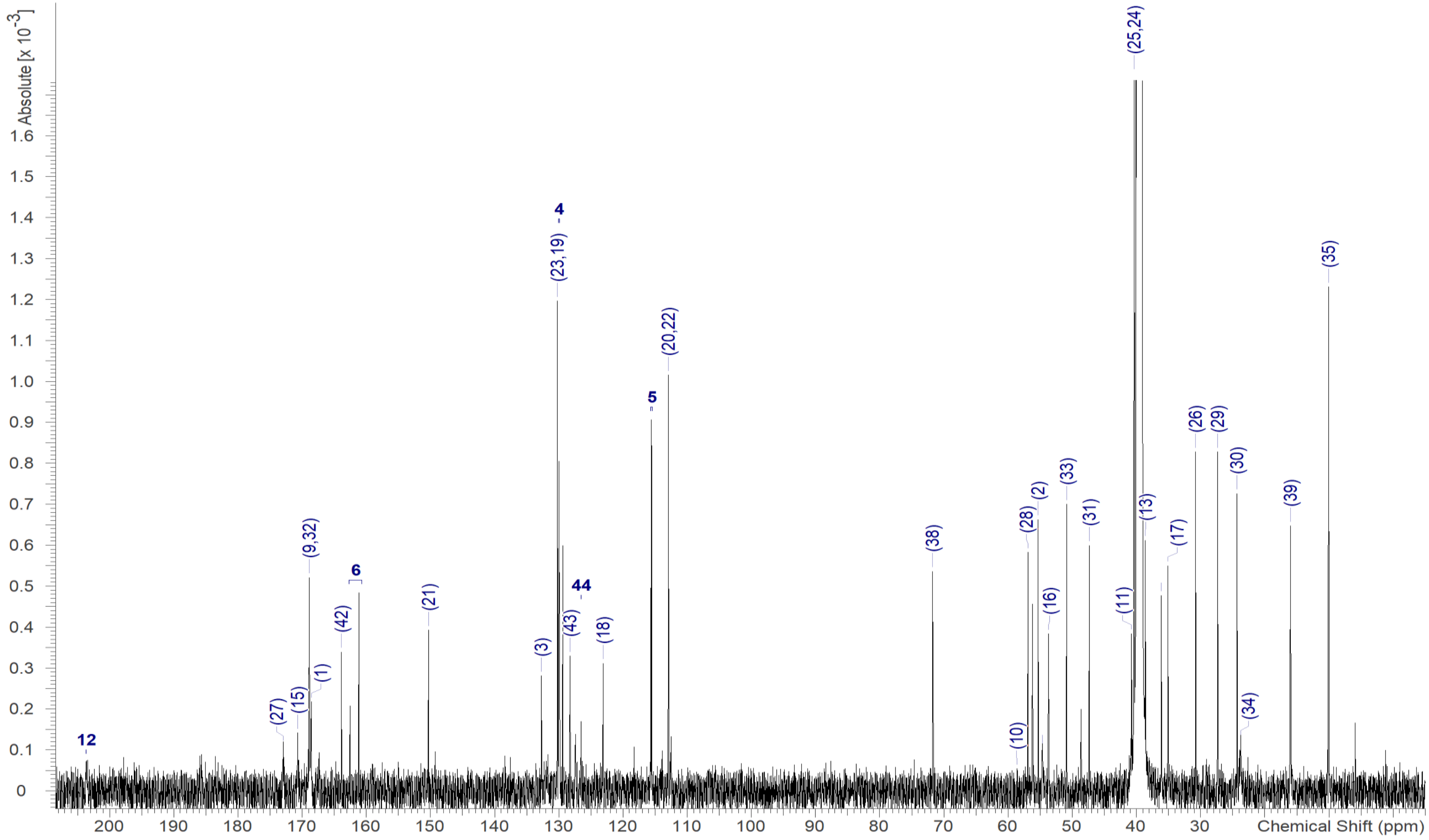

Figure S17: ^{13}C NMR spectrum (700 MHz) 6-fluoropristinamycin I (**4**) in DMSO-d_6.

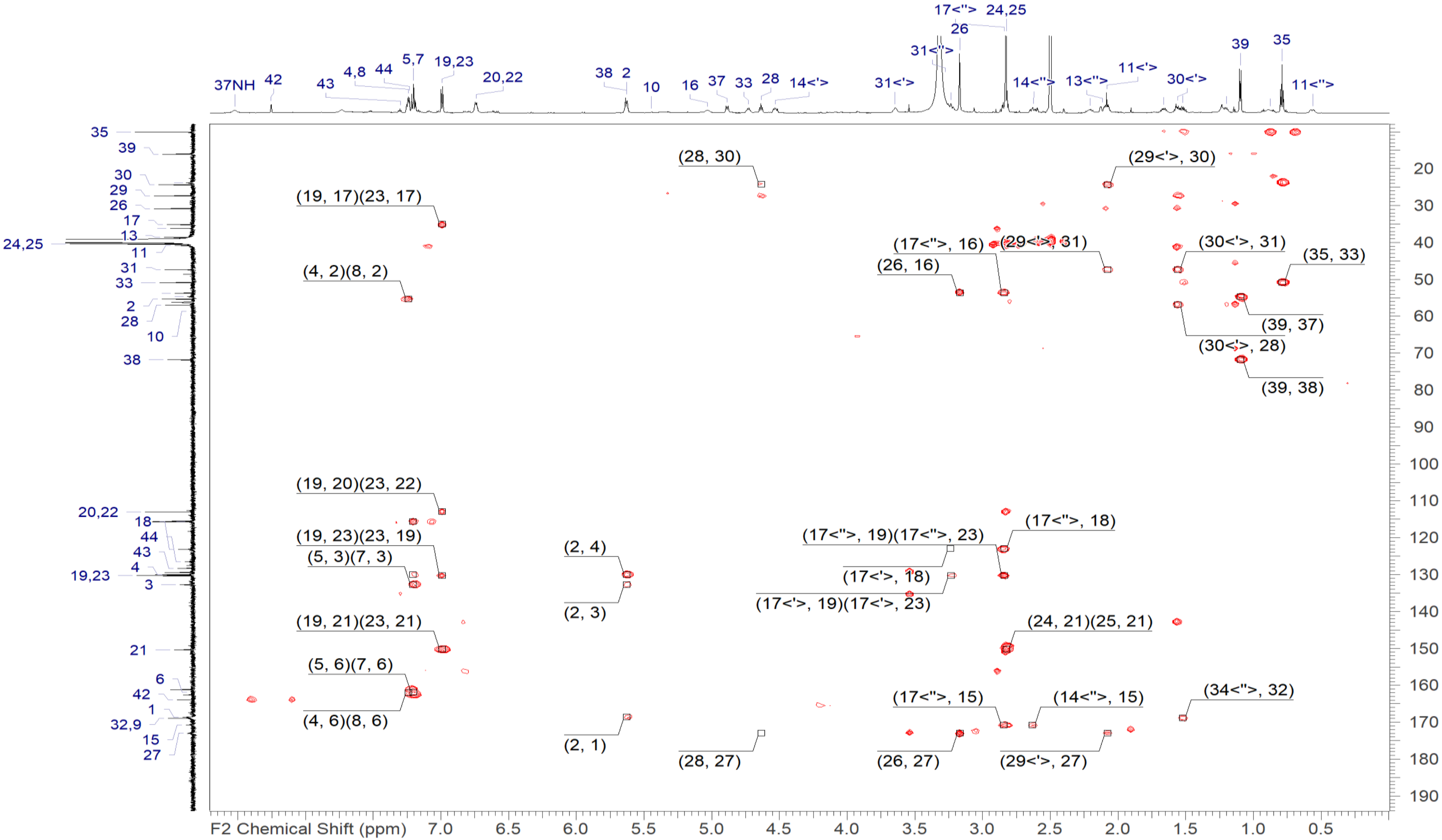

Figure S18: HMBC spectrum (700 MHz) 6-fluoropristinamycin I (**4**) in DMSO-d_6.

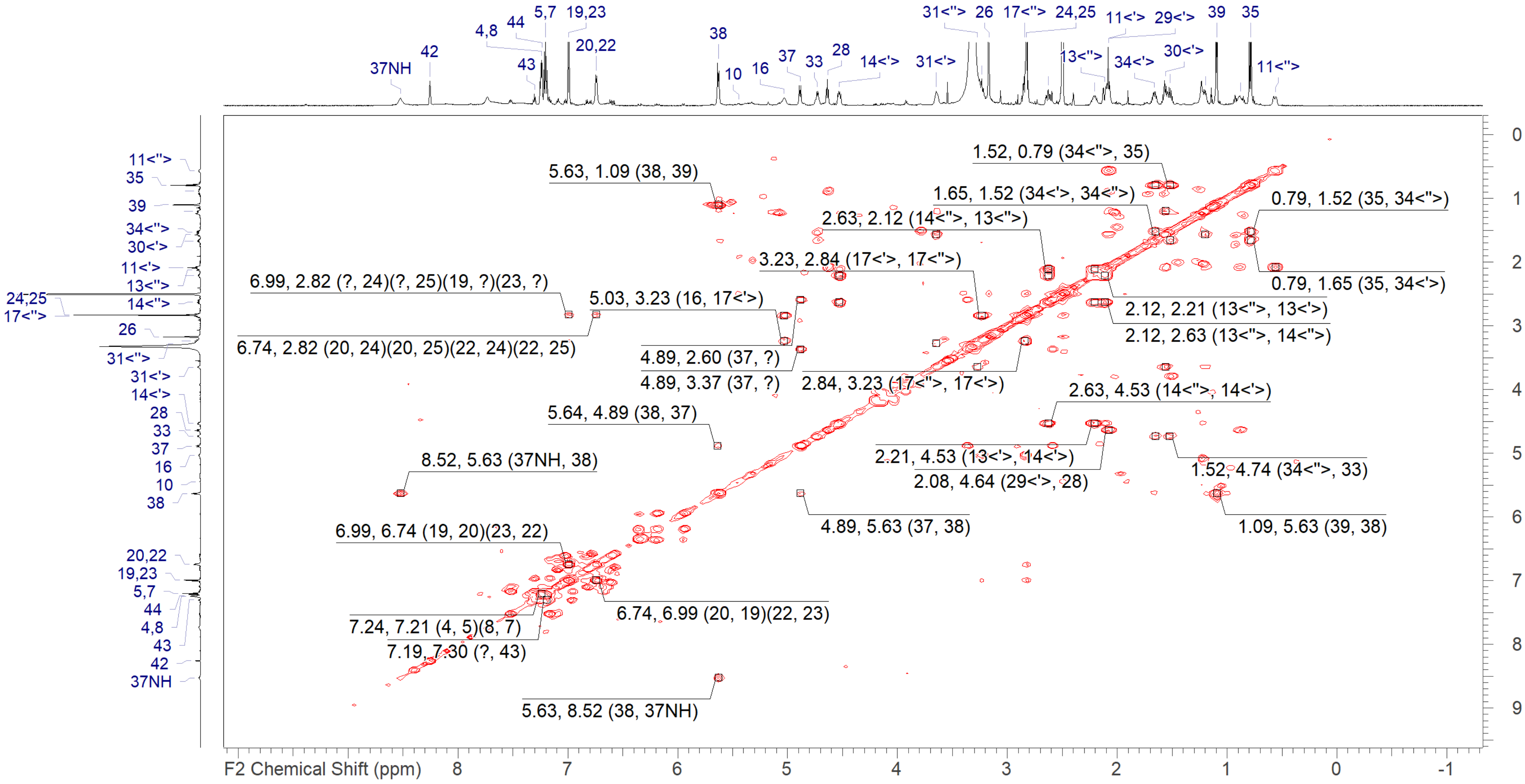

Figure S19: COSY spectrum (700 MHz) 6-fluoropristinamycin I (**4**) in DMSO-d_6.

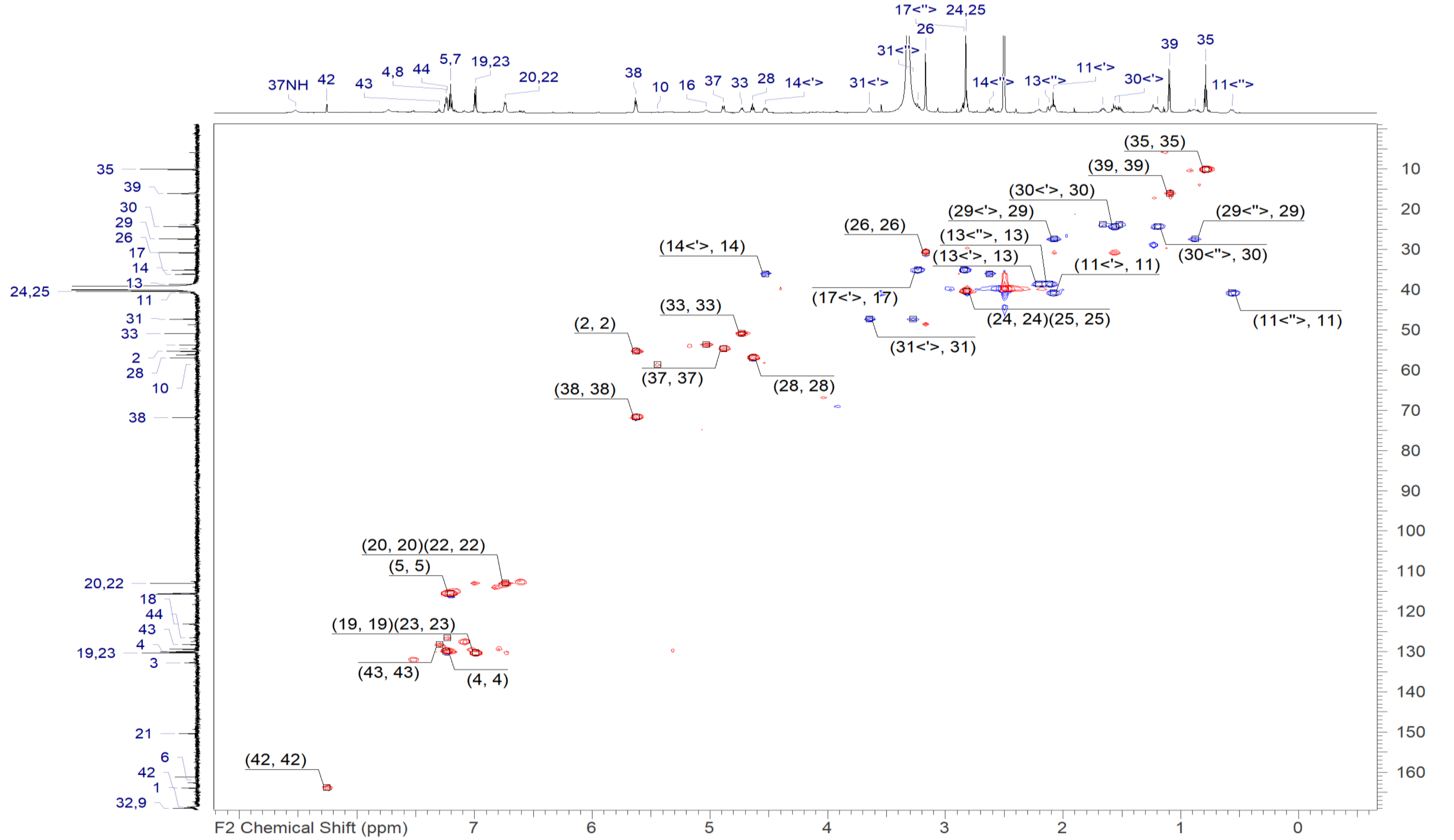

Figure S20: HSQC-DEPT NMR spectrum (700 MHz) 6-fluoropristinamycin I (**4**) in DMSO-d_6.

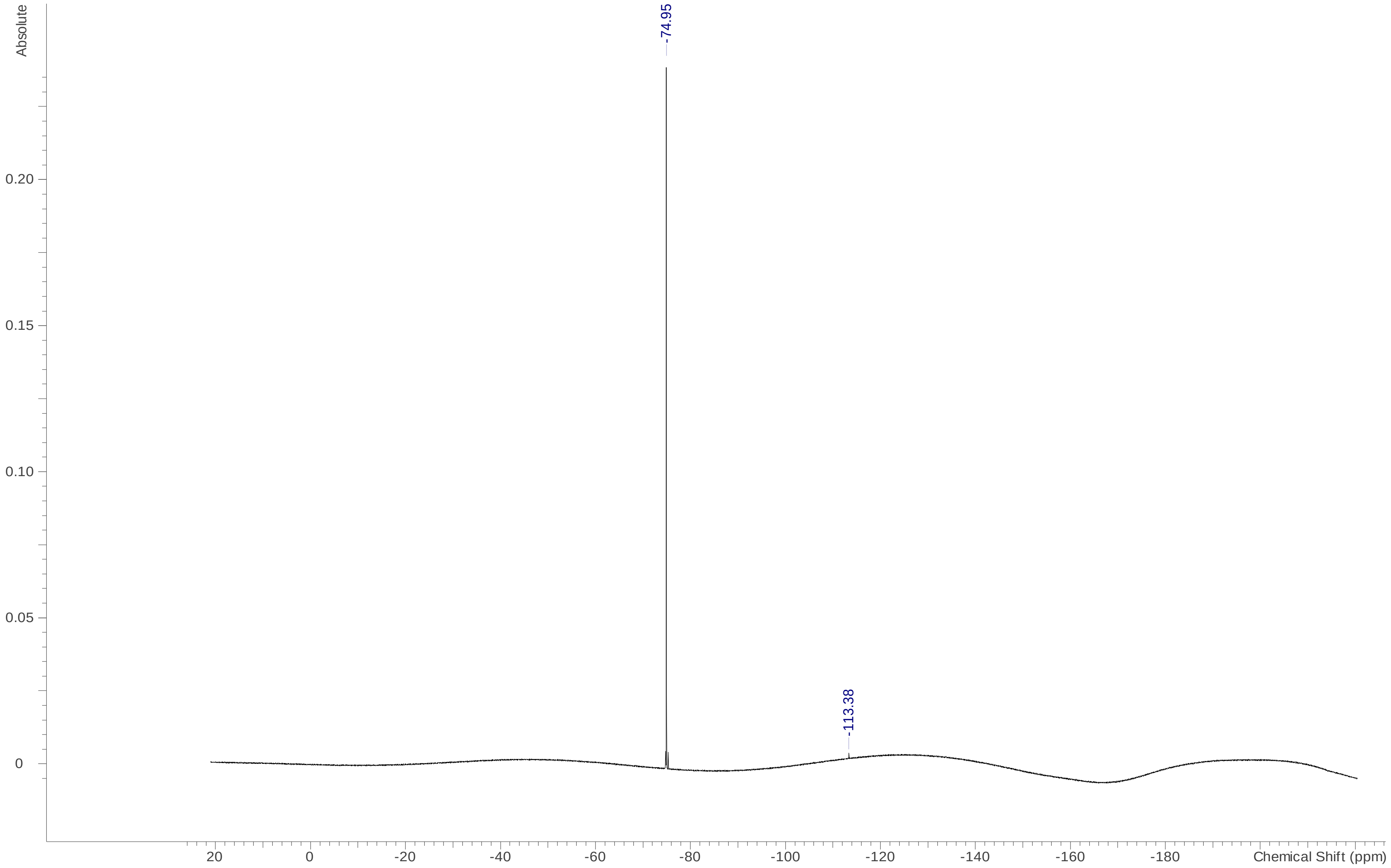

Figure S21: ^{19}F NMR spectrum (470 MHz) 6-fluoropristinamycin I (**4**) in DMSO-d_6.

A *E. faecium* DSM 20477

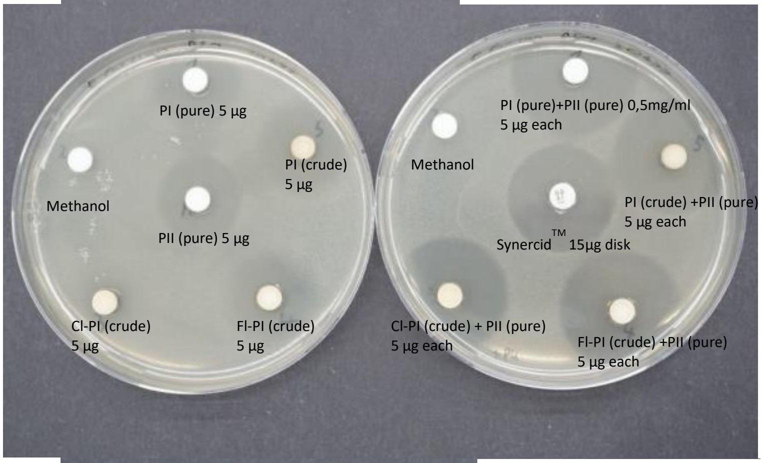

B *S. aureus* DSM 18827

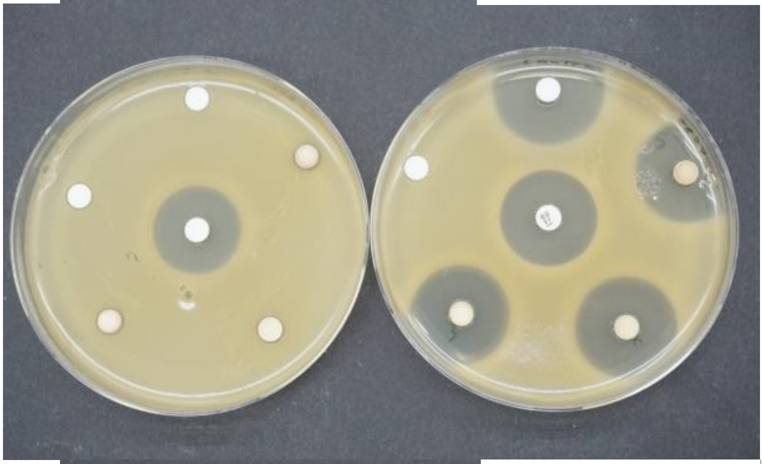

C *S. aureus* CIP 111304

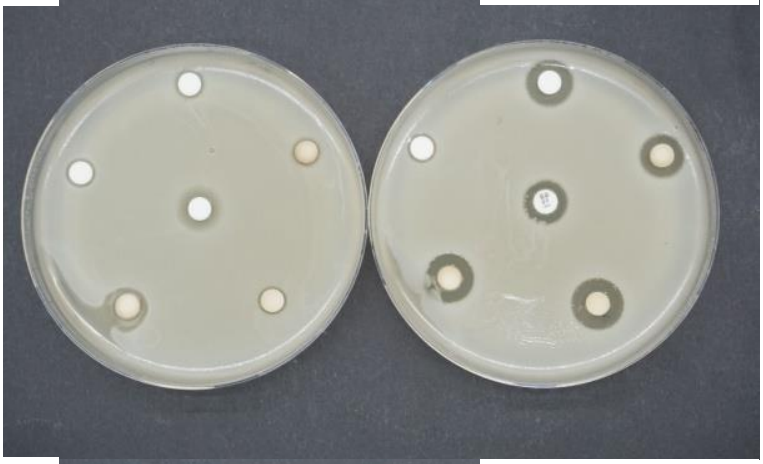

D *S. aureus* CIP 108540

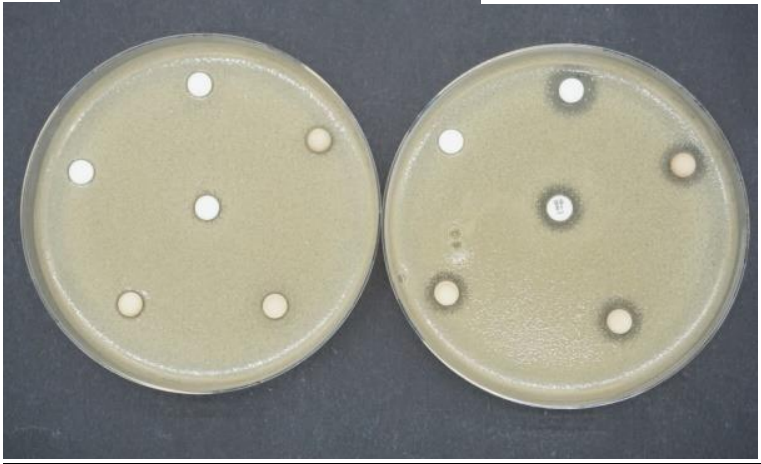

Figure S22: Results of the agar diffusion test of PI derivatives alone and in combination with PII against streptogramin susceptible clinical isolates. The pattern of sample application follows that of the *E. faecium* DSM 20477 example.

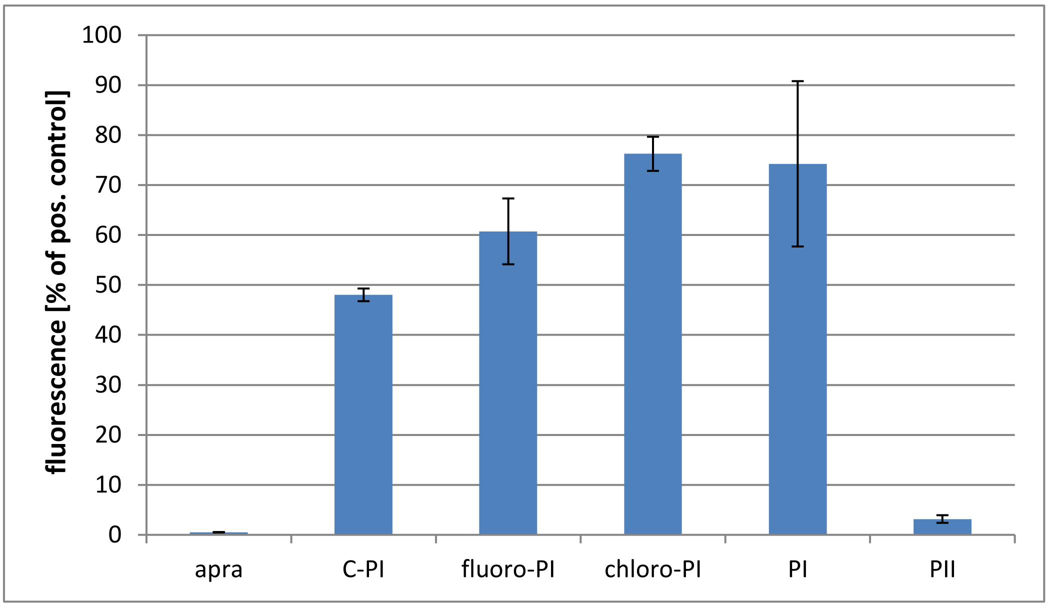

Figure S23: Results of the *in vitro* transcription translation assay used to investigate the effect of semi purified PI derivatives as well as pure pristinamycin I (PI) and II (PII) on bacterial protein biosynthesis. Apramycin (apra) served as a positive control (C-PI = PI isolated from *S. pristinaespiralis ΔpglAΔsnaE1* supplemented with L-Phg).

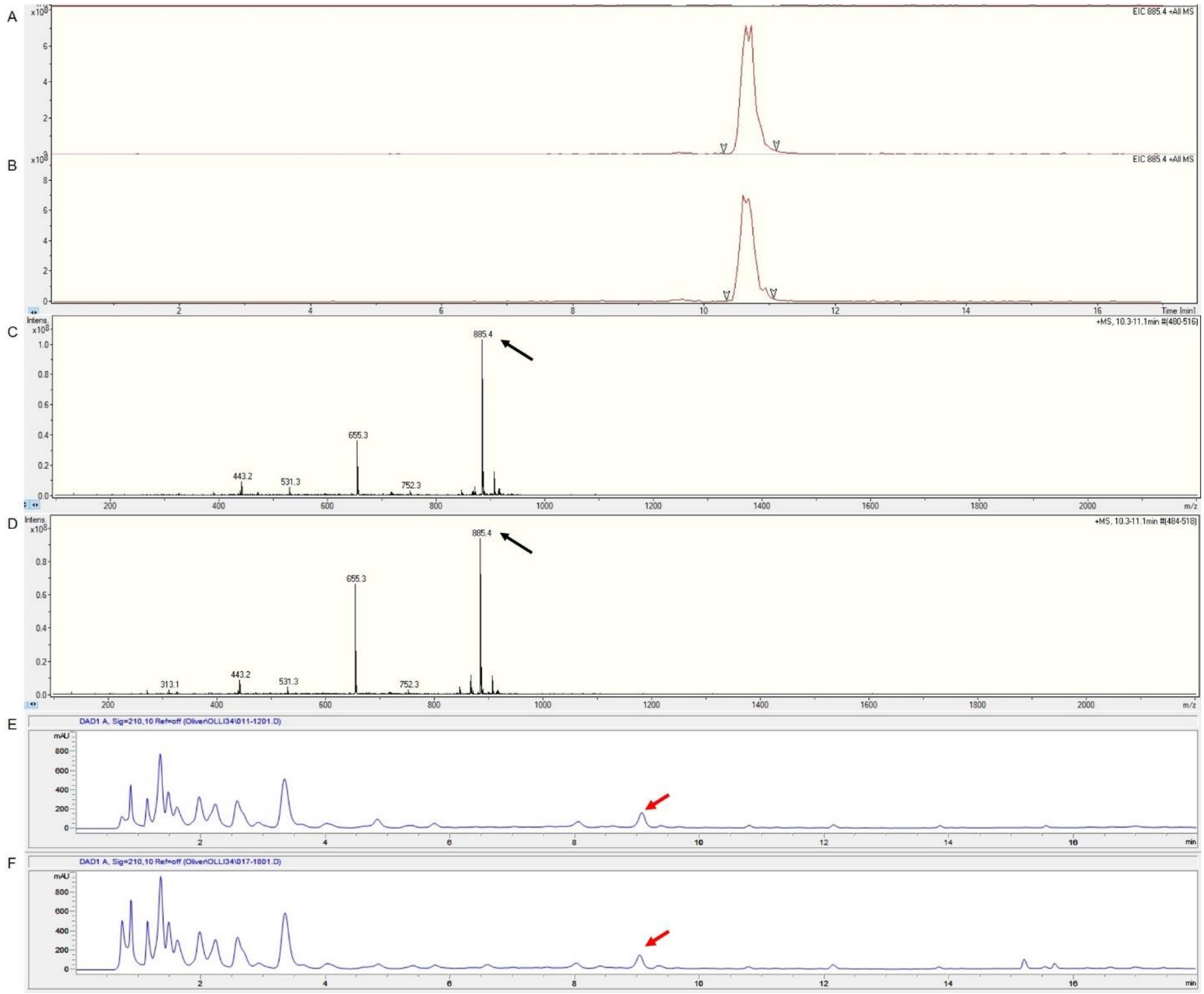

Figure S24: HPLC/MS analysis of *S. pristinaespiralis ΔpglAΔsnaE1* extracts from cultures supplemented with pure 4-fluoro-Phg (A, C, E) and 4-fluoro-Phg containing *E. coli* BL21(DE3) pET28-*hmo*/pACYC-*bcd-gdh* supernatant (B, D, F), respectively. A and B are the extracted ion chromatograms (EIC) for the mass corresponding to 6-fluoropristinamycin I (**4**) (*m/z* 885.4 $[M+H]^+$) in positive mode. C and D are the corresponding mass spectra. E and F are separate UV chromatograms at 210 nm (at the same scale). The mass signals for **4** are marked with black arrows and the respective UV signals are marked with red arrows.

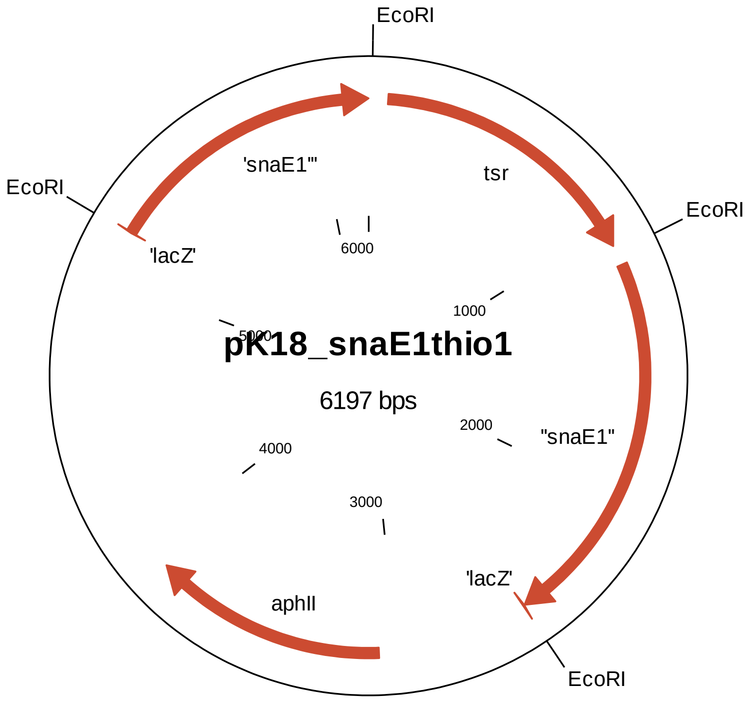

Figure S25: Cloning chart of mutagenesis plasmid pK18/snaE1tsr.

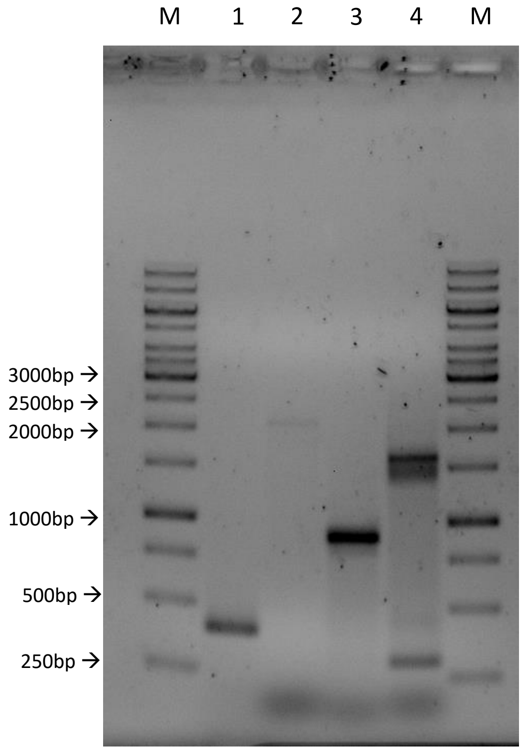

Figure S26: Verification of the *snaE1* mutation by PCR. Amplificates are shown for PCR with primer pairs thio1/2 (lane 1; ≈0.4 kb), KnsaE1fw/rv (lane 2; ≈2.1 kb), KnsaE1fw/thio2 (lane 3; ≈0.8 kb), and KnsaE1rv/thio1 (lane 4; ≈1.7 kb) separated in a 1% agarose gel. Marker (M): 1 kb length marker, Thermo Fisher Scientific.

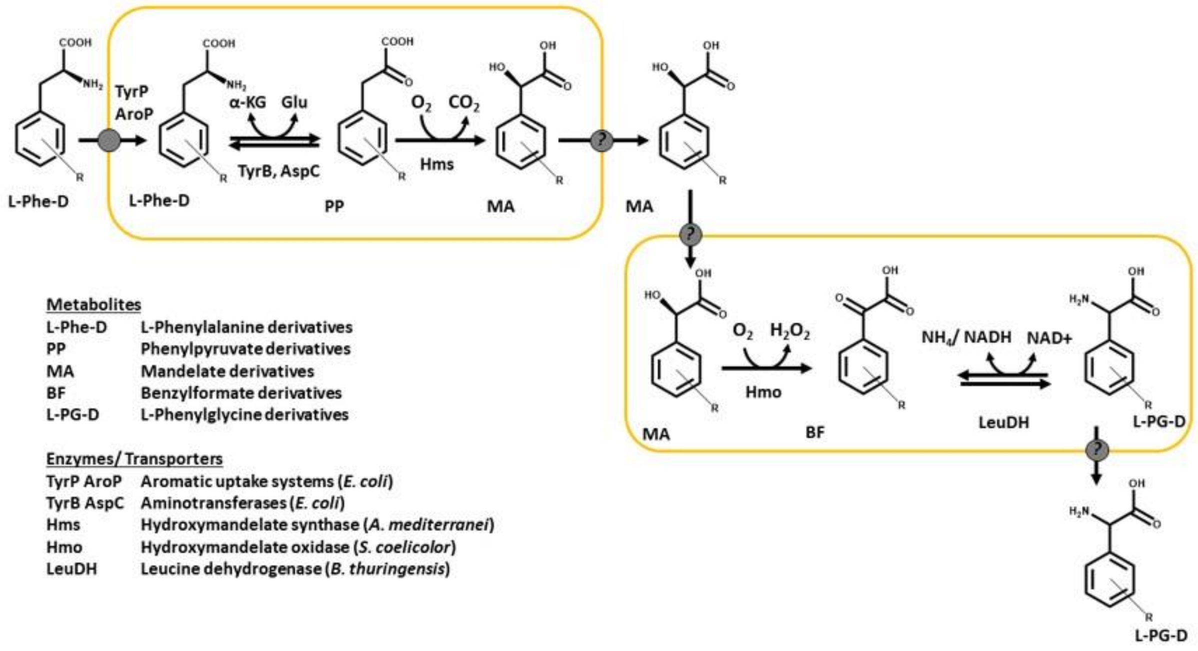

Figure S27: Schematic representation of the biotransformation approach for the synthesis of L-Phg derivatives including respective enzymes and metabolites involved in each step.

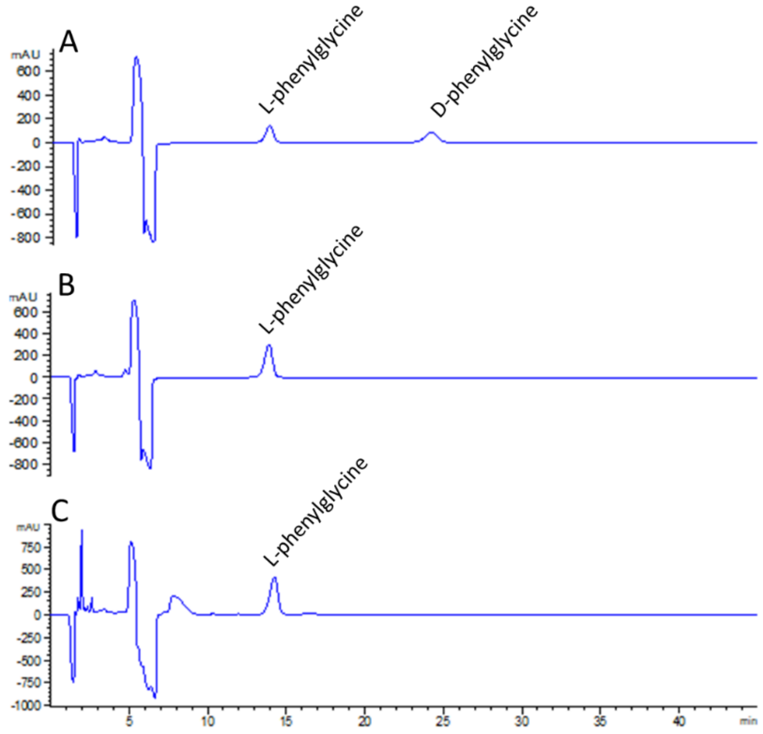

Figure S28: Representative chiral HPLC chromatogram of the separation of Phg enantiomers at 210 nm. Chromatogram A and B illustrate the separation of commercially available standards for DL-Phg and L-Phg. C is showing a typical chromatogram of the whole cell biotransformation approach with *E. coli* BL21(DE3) pET28-*hmo/* pACYC-*bcd-gdh* with L-mandelic acid.

References

1. Y. Mast, W. Wohlleben, E. Schinko, Identification and functional characterization of phenylglycine biosynthetic genes involved in pristinamycin biosynthesis in *Streptomyces pristinaespiralis. J. Biotechnol.*, 2011, **155**, 63-67.
2. D. Moosmann, V. Mokeev, A. Kulik, N. Osipenkov, S. Kocadinc, R. Ort-Winklbauer, F. Handel, O. Hennrich, JW. Youn, GA. Sprenger, Y. Mast, Genetic engineering approaches for the fermentative production of phenylglycines. *Appl Microbiol Biotechnol*, 2020, **104**, 3433–3444.
3. RD. Pridmore, New and versatile cloning vectors with kanamycin-resistance marker. *Gene*, 1987, **56**, 309-312.
4. J-W. Youn, C. Albermann, G. A. Sprenger, *In vivo* cascade catalysis of aromatic amino acids to the respective mandelic acids using recombinant *E. coli* cells expressing hydroxymandelate synthase (HMS) from *Amycolatopsis mediterranei*. *Molecular Catalysis*, 2020, **483**, 110713.
5. A.C. Chang, S.N Cohen, Construction and characterization of amplifiable multicopy DNA cloning vehicles derived from the P15A cryptic miniplasmid. *J Bacteriol.,* 1978, **134**, 1141-1156.
